# Biological Effects of Magnetic Storms and ELF Magnetic Fields

**DOI:** 10.3390/biology12121506

**Published:** 2023-12-08

**Authors:** Ruslan M. Sarimov, Dmitry A. Serov, Sergey V. Gudkov

**Affiliations:** Prokhorov General Physics Institute of the Russian Academy of Sciences, 38 Vavilova Street, 119991 Moscow, Russia; rusa@kapella.gpi.ru (R.M.S.); dmitriy_serov_91@mail.ru (D.A.S.)

**Keywords:** magnetobiology, geomagnetic field, extremely-low-frequency magnetic fields, cardiovascular system, leukemia

## Abstract

**Simple Summary:**

The study of the biological effects of time-varying magnetic fields has attracted more and more attention from researchers, and the number of publications on this topic is growing every year. In this article, we plan to briefly introduce the reader to the results of research, ideas, and discussions on the biological effects of time-varying magnetic fields. This article is illustrated with a large number of generalizing figures and contains a lot of factual data. This review presents the main biological effects observed during magnetic storms and in laboratory studies. The general concepts of studying the influence of magnetic storms on humans are described. Possible approaches to modeling magnetobiological effects at different levels of the organization of living things are presented. The results of the impact of anthropogenic fields on humans (epidemiological studies) are presented. The mechanisms of action of time-varying magnetic fields on living objects are discussed. Dependences of quantitative characteristics of the biological action of time-varying magnetic fields on their frequency, induction, and duration are discussed. The information presented in this manuscript may be valuable for a wide range of readers in the initial assessment of the risks associated with the influence of time-varying magnetic fields on the body.

**Abstract:**

Magnetic fields are a constant and essential part of our environment. The main components of ambient magnetic fields are the constant part of the geomagnetic field, its fluctuations caused by magnetic storms, and man-made magnetic fields. These fields refer to extremely-low-frequency (<1 kHz) magnetic fields (ELF-MFs). Since the 1980s, a huge amount of data has been accumulated on the biological effects of magnetic fields, in particular ELF-MFs. However, a unified picture of the patterns of action of magnetic fields has not been formed. Even though a unified mechanism has not yet been generally accepted, several theories have been proposed. In this review, we attempted to take a new approach to analyzing the quantitative data on the effects of ELF-MFs to identify new potential areas for research. This review provides general descriptions of the main effects of magnetic storms and anthropogenic fields on living organisms (molecular–cellular level and whole organism) and a brief description of the main mechanisms of magnetic field effects on living organisms. This review may be of interest to specialists in the fields of biology, physics, medicine, and other interdisciplinary areas.

## 1. Introduction

The geomagnetic field (GMF) is a global vector field with an induction of 25–65 μT, depending on proximity to the Earth’s magnetic poles [1,2]. The GMF consists of a constant and a varying component. Variations in the GMF compared to constants usually do not exceed 1–5% and are caused by electric current systems in the Earth’s ionosphere [3,4,5,6]. Even for a long time (~10 years) GMF induction fluctuations do not exceed 1–2 µT [7]. The GMF plays a key role in ensuring life on Earth, in a sense, along with oxygen and water [6,8,9]. The GMF performs several functions that ensure the presence of life on Earth: it protects the atmosphere from the loss of oxygen, hydrogen, and other light elements due to the solar wind [10,11,12,13], preserves the integrity of the ozone layer, contributes to maintaining a constant climate on Earth, serves as a guide for the migration of birds and animals, and participates in the regulation of circadian rhythms in plants and animals [14,15,16,17,18]. Perhaps the presence of the GMF was one of the conditions for the beginning of abiogenesis and the emergence of “chiral purity” of living beings [19,20,21,22,23].

**Figure 1:**
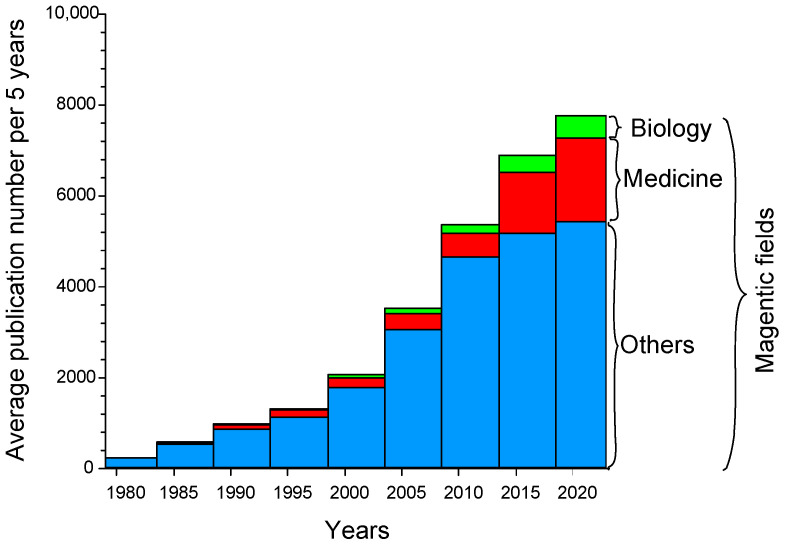
The dynamics of the number of publications containing the keywords “magnetic field” (all bars), “magnetic field medicine” (red), and “magnetic field biology” (green). Other works are indicated in blue. Data taken from PubMed database (https://pubmed.ncbi.nlm.nih.gov/, accessed on 15 October 2023).

Given the above, magnetic fields (MFs) play an important role in the life of humans and other inhabitants of the Earth. The number of publications devoted to the study of MFs has been growing from the 1980s to the present (Figure 1). The proportion of works devoted to the medical aspects of MF applications and their biological effects has significantly (several times) increased over the past 10 years.

In addition to the GMF and its fluctuations, a human is constantly exposed to urban MFs generated by electrical networks and transport [24]. Therefore, the biological effects of low-frequency, time-varying magnetic fields (TVMFs) are important [3,4,25].

Geomagnetic storms cause GMF induction fluctuations with frequencies from 0.00007 to 30 Hz and amplitudes of fluctuations from 70 to 900 nT depending on latitude, but more often they do not exceed 200 nT [26,27,28,29,30]. It is worth noting that despite the low amplitudes, the biological effects of magnetic storms are significant [24]. A possible explanation is a relatively long exposure (hours), but it is not exhaustive [24,28].

In addition to the GMF, the main background TVMF is the field generated by power lines, having a frequency of 50 or 60 Hz and induction fluctuations of ~0.05 to ~2.6 μT and higher [31,32,33,34,35]. Workers in industrial production and railway transport are exposed to TVMF with an induction of 0.3–2.5 μT [30]. The TVMF induction near high-voltage power lines and transformer stations is up to 20 μT for 380 kV and up to 400 nT for 15 kV [31,34].

Semiconductor factory workers are exposed to ELF-MF with an induction of 15–35 μT [34,36]. A TVMF frequency generated from road transport and within a city varies between 10^−3^ and 10^2^ Hz [24]. It is noteworthy that in the frequency range of 10^−3^–1 Hz the TVMF induction generated within a city and from transport is higher than the amplitude of variation in the GMF during a strong (k = 8) magnetic storm [24].

A significant number of works, including early ones (1980s–1990s), are devoted to the study of the biological effects of microwaves and electromagnetic waves of 0.3–300 GHz [37,38,39,40]. However, in this case, fundamentally different methodological and metrological approaches are used [41,42,43,44,45], and the array of data is so large that it is not possible to analyze the effects of low-frequency (<some kHz) and high-frequency (>1 MHz) MFs in sufficient detail in a single article. Recent studies indicate that mobile phones can generate extremely-low-frequency magnetic fields (ELF-MFs) within a frequency range of 5–200 Hz. The magnetic induction of the ELF-MF generated during mobile phone emission can be as high as 70–80 μT [46,47]. Consequently, comprehending the biological implications of exposing oneself to ELF-MFs is pivotal to understanding the potential long-term effects of prolonged mobile phone usage.

This review will be devoted to extremely low-frequency magnetic fields (<1 kHz; ELF-MFs) [48,49,50]. Firstly, ELF-MFs include the city fields and GMF disturbances during magnetic storms. Secondly, there are many differences between the methodology of ELF-MF experiments and the study of EMFs in the megahertz (LTE) and gigahertz (5G) frequency ranges [37,38,39,40]. For example, for EMFs with frequencies >150 MHz or >6 GHz, the design wavelength will be <2 m or <2 cm. In this case, the EMF becomes spatially inhomogeneous, especially for the GHz range. Therefore, a significant variation in the degree of magnetic influence is possible even for samples placed in rows within the same experiment. For example, the calculated wavelength is >29 km for frequencies below 1 kHz. In this case, the spatial distribution of the EMF within the facility is easier to characterize and predict.

Despite the established biological effects of ELF-MFs [51], the mechanisms of their biological effects remain unclear [52]. The energy of GMFs or anthropogenic ELF-MFs is much less than the activation energy of chemical reactions *kT* (where *k* is Boltzmann’s constant and *T* is absolute temperature). Therefore, thermal effects on the elementary act of chemical reactions are excluded [53]. At the same time, the biological effects of ELF-MFs are often described in the literature [48]. Moreover, these effects can manifest themselves at certain (rather narrowly localized) values of the frequency and amplitude of ELF-MFs and be absent at other frequencies and amplitudes of the same order [42,54,55,56,57]. Over the past few decades, several hypotheses have been proposed in this regard. However, there is no definitive understanding of the formation of a biological response to ELF-MFs.

This review describes the main directions of reactions of biological systems to ELF-MFs, provides an excursion into the main mechanisms of the biological action of MFs, and attempts to systematize literature data to search for new patterns of connection between the amplitude of the biological response and the amplitude–frequency characteristics of ELF-MFs.

The total number of works based only on NCBI PubMed data (https://pubmed.ncbi.nlm.nih.gov/ assessed on 15 October 2023) with the keywords “magnetic field” + “biology” or “magnetic field” + “medicine” exceeds 2400 and 7100 works, respectively. The term ELF-MF is explicitly mentioned in several hundred papers on both fronts. In other databases, the number of publications is expected to be higher. We understand that it is impossible to review the entire variety of works in this area within the framework of one article, so we included only part of these works in the present review. We attempted to include papers from different publication years containing data on different biological effects of ELF-MFs. It is worth noting the significant differences between the “quality” of publications on this issue. Therefore, before inclusion in this review, we checked the works according to several criteria.

The criteria for selecting articles to evaluate the magnetobiological effects of ELF-MFs were the presence of adequate sham controls, a description of the type of installation, and its operating mode. The implementation of sinusoidal variable fields also emerged as one of the primary preferred selection criteria. To assess the quality of the publication, we selected four parameters:

(1) The use of adequate methods of statistical analysis (ANOVA, ranks, or parametric tests after checking their applicability);
(2) A detailed description of the ELF-MF’s characteristics and an assessment of its homogeneity within the experimental setup (preferably, the presence of a 3D map of the spatial distribution of induction during the experiment);
(3) The availability of instrumental verification of the parameters of the surrounding MF, measures to compensate (if necessary) for the installation for generating the MF, and possible sources of artifacts (background fields, field inhomogeneity in the installation);
(4) The SJR rating of the journal in which the work was published, as a measure of the relevance of the work as a whole (we chose the threshold SJR > 0.4).

Exclusion is based on not agreeing with one or more of the specified above criteria. In the case of laboratory and epidemiological studies, detailed data are given below.

## 2. Biological Effects of Magnetic Storms

### 2.1. Approaches to Research

Human health is a main object in this scientific area. Two general approaches can be used to study the influence of GMF fluctuation effects on a human:

(1) Analysis of a large array of data: physiological, usually clinical, and data on geomagnetic activity [29].
(2) Simulation of geomagnetic storm conditions in the laboratory and the monitoring of physiological parameters of volunteers [28,58].

Data on geomagnetic activity are publicly available, and the researcher chooses the level of detail of their analysis based on his task. Clinical data are not open. However, subject to all ethical and confidentiality standards, their analysis is possible for scientific purposes. As a rule, researchers operate with metadata with a limited number of characteristics to optimize the analysis procedure and specify patterns. The advantages of this approach are the following:

Firstly, researchers have the opportunity to work with very large (thousands and tens of thousands) samples of “subjects” [59]. This allows them to obtain results with a high degree of accuracy and statistical significance. The researcher has the opportunity to analyze both mass cases (ischemic diseases, heart attacks, and strokes) [59], and individual groups of people differing in age, gender, and place of residence [60,61].

Secondly, geomagnetic activity data is recorded and stored centrally, as well as clinical metadata, so the results of their analysis will be very reproducible.

Third, metadata collected over time can be represented as a long time series with high temporal resolution. In this case, automated spectral analysis methods can potentially be applied to them: wavelet transforms, cross-correlation assays, bispectral analyses, etc. [62,63]. The use of neural networks and AI technologies may expand the capabilities of this analytical approach.

A separate sub-item of this approach can be considered the analysis of metadata of patients and/or behavioral reactions of large populations of animals under conditions of different anthropogenic loads of TVMFs [32,64]. The effects of background MFs will be discussed in more detail below.

The disadvantages of this approach are listed below.

Firstly, most works use integral indicators of the induction and frequency values of the GMF during a magnetic storm [65,66,67]. Obtaining detailed amplitude–frequency characteristics will allow for the acquisition of additional information about the possible mechanisms of MF effects on biological functions [52]. It is often not possible in the case of publicly published geomagnetic data.

Secondly, the time detail of data on GMF state from publicly available sources cannot exceed 3 h, for technical reasons (https://www.swpc.noaa.gov/products/planetary-k-index, accessed on 22 September 2023, https://xras.ru/magnetic_storms.html, accessed on 22 September 2023, https://sunearthday.nasa.gov/swac/tutorials/mag_kp.php, accessed on 22 September 2023). In addition, in these works the assessment is carried out using integral indicators during the day to save computing power [61,62,68]. All of the above makes it difficult to track the times of “impact” and “effect”. The way out of this situation is to combine independent measurement of the MF spectral content on the days of interest for GMF disturbances and the collection of metadata about patients and volunteers on specific dates. The approach is more labor-intensive but possibly will allow the use of more accurate data analysis methods.

Third, volunteer or patient data completion may significantly vary across countries, complicating analyses when combining data from multiple studies. Often researchers have to limit themselves to certain periods and regions [69,70]. Such studies are fundamentally impossible in regions without an established level of standardization of medical documentation.

Fourthly, the collection of geomagnetic disturbances occurs mainly in heliophysical observatories, and the recording of the bioeffects of geomagnetic disturbances occurs mainly for residents of cities: firstly, remote from these observatories [29,59], and secondly, against the background of the magnetic noise of the city [24].

The lack of detailed amplitude–frequency and temporal characteristics of GMF fluctuations does not allow the use of this approach to study possible mechanisms of MF action. On the other hand, a powerful statistical base and high reproducibility make it possible to obtain practically useful data of an applied nature. The latter is the reason why geomagnetic monitoring is used to predict the health status of a meteosensitive part of the population.

Simulation in the laboratory consists of creating TVMFs with a spectral content close to GMF disturbances of a given magnitude with a special device. MF generators are systems of coils, usually Helmholtz systems, sometimes with additional shielding of the external electric field (Faraday grid) [58,71].

This approach has the following advantages:

Firstly, the possibility of obtaining TVMF oscillations with precisely specified spectral content. In conjunction with continuous and long-term recordings of physiological parameters, this makes it possible to assess the relationship between physiology and the characteristics of GMF fluctuations. This approach gives more accurate time frames for time to effect, allows for the performance of complex and accurate methods of mathematical analysis to assess correlations between GMF induction oscillations and physiological responses of organisms, allows for the search for resonance phenomena in the living systems, etc. [58,71,72,73,74,75]. With the collection of sufficient statistical material, it will become possible to analyze the fundamental mechanisms of the interaction of MFs with living systems [76,77].

Secondly, the ability to add new, additional conditions; for example, the influence of microgravity [71].

Thirdly, the set of measured parameters can be adapted to the specific research task. The same equipment is used for data recording within all series of experiments. External conditions (light, temperature, etc.) are standard. Raw data are received by one team of employees. All this is intended to increase the reproducibility of results, even for small samples [28].

The disadvantages of this approach include:

Firstly, research is highly labor-intensive and costly. Unlike “classical” systems (Ø 10–150 cm), the dimensions of TVMF exposure systems for experiments on volunteers are several meters [28,58,71]. It is also necessary to create comfortable conditions for the subject and maintain their consistency.

Secondly, a consequence of the first is that the samples in these studies usually do not exceed a dozen people [28]. This limits the scope of application of the approach in medicine and allows magnetic storm modeling to be used only for fundamental research.

Thirdly, there are few works on the active modeling of GMF disturbances, and the installations used in them, as a rule, are unique for each group of authors [78]. These conditions significantly complicate the analysis and averaging of results on this topic.

### 2.2. Biological Effects

**Figure 2:**
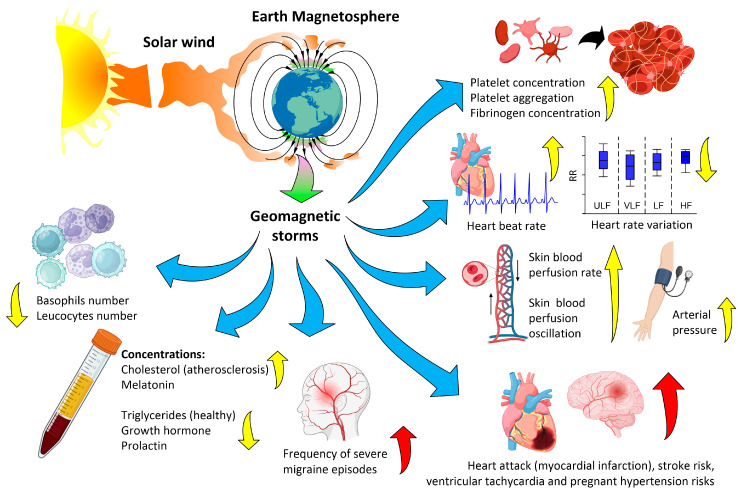
The main biological effects of magnetic storms on an organism are described in the literature (references can be found in Table 1). The up and down arrows indicate an increase or decrease in a parameter, respectively. The color indicates the expected impact of the effect on the organism under study: red—changes assessed by the authors of the original work as negative, yellow—difficult to unambiguously assess.

The main directions of the biological effects of magnetic storms are shown in Figure 2. Specific examples are given in Table 1. Most of the works devoted to the study of the influence of GMF disturbances on the human body describe the effects on the circulatory system. This is due to several of reasons: a large number of metadata, the technical ability to monitor the condition (Holter monitoring), and probably the high sensitivity of this body system to GMF disturbances [58,59]. These effects can be divided into groups according to the level of organization: individual blood cells, blood vessels, and the state of the heart in normal and pathological conditions [79,80,81]. Magnetic storms affect blood clotting; in particular, they increase platelet concentration, prothrombin time, platelet aggregation, and fibrinogen concentration [82,83,84]. On the other hand, a decrease in basophil and leukocyte numbers was shown during magnetic storms [67]. Effects at the molecular level include a decrease in the concentration of cholesterol (with atherosclerosis) and triglycerides (healthy) in the blood [85] and an increase in the concentrations of growth hormone and prolactin [86]. Magnetic storms affect both the micro- and macrocirculation in the bloodstream.

First, GMF disturbances cause an increase in capillary blood flow rate and the average time of capillary closure [28,84]. In addition, magnetic storms affect the dynamics of the speed of capillary blood flow. Periodic changes in the speed of skin microcirculation are a very sensitive marker of the physiological state of the body under normal conditions, with age-related changes and pathology [87,88,89,90,91,92]. The effects on microcirculation consist of an increase in the amplitude of oscillation of skin blood flow rate in response to magnetosphere disturbances [62]. A significant correlation of skin microcirculation oscillation with low-frequency oscillations of GMF induction at frequencies of ~0.01, ~0.03, ~0.1, and 0.3 Hz has been shown [62]. It is very informative to assess the degree of correlation between microcirculation fluctuations in different rhythms. This approach can be used for non-invasive techniques for diagnosing and monitoring the development of diabetes mellitus, bronchial asthma, and other pathologies [90,91,93,94,95,96,97]. The use of a correlation approach to the study of microcirculation oscillations in different rhythms during a magnetic storm may open new aspects of the physiological effects of weak ELF-MFs in the future.

Macrocirculation changes depend on changes in blood pressure, absolute heart rate, and heart rate variability. Magnetic disturbances and storms can lead to an increase in the average daily heart rate observed during [98], and a decrease in the amplitude of heart rate variability in, the low-frequency (LF) interval [99]. A weakening of heart rate variability in almost all frequency ranges has been shown during the simulation of a magnetic storm [78]. A high correlation of heart rate variability parameters with GMF induction oscillations and solar wind speed has been shown [29]. Significant changes in heart rate variability in low-frequency ranges may indicate the occurrence of arrhythmia [100,101,102,103,104]. A systolic and diastolic blood pressure increase is observed during a magnetic storm. It is probably caused by an increase in heart rate [78,98,105]. The intra-annual dynamics of the incidence of cerebral and coronary vascular accidents are uneven and have an oscillatory, cyclical nature. They reliably correlate with the dynamics of solar flare activity and geomagnetic activity. The incidence of myocardial infarction correlates to a greater extent with geomagnetic activity, while the incidence of cerebral strokes correlates with solar activity [106]. An increased load on the heart leads to an increased risk of exacerbation of diseases of the cardiovascular system: myocardial infarction, stroke, ventricular tachycardia, and hypertension in pregnant women [59,68,107,108,109]. Hemoglobin and hematocrit concentrations do not change under the influence of geomagnetic storms [67,110]. As a consequence, an increase in the load on the cardiovascular system during magnetic storms is caused not by a change in oxygen capacity but by viscosity due to changes in systemic blood coagulation [82,83,84]. Increases in heart rate and blood pressure are designed to compensate for the rate of blood transport, which in turn increases the risk of heart failure and mortality from these disorders [59,98].

A connection was found between the frequency of recorded episodes of moderate and severe migraine and the presence and integral induction of geomagnetic disturbances [66]. Magnetic storms change the redistribution in the activity of parts of the autonomic nervous system: increasing the contribution of the parasympathetic part and reducing the contribution of the sympathetic part [98]. A connection between geomagnetic disturbances and behavior and well-being has been discovered [99]. A connection between strong magnetic storms and an increase in the frequency of suicides has been shown [111]. High levels of background geomagnetic activity in northern latitudes (>80 nT) significantly reduce the daily synthesis of melatonin, which can disrupt circadian rhythms [60].

Many factors of both cosmic and terrestrial origin change during geomagnetic storms. In addition to the flux of charged particles reaching the Earth’s surface, the correlations of geomagnetic field fluctuations with atmospheric pressure [112] or electric field [113] are known. However, these geophysical parameters also vary independently of the geomagnetic disturbances. For example, these changes are more pronounced during thunderstorms. Therefore, when describing the magnetobiological effects of magnetic storms in the article, the emphasis is placed on the magnetic component of such effects. Moreover, there are experimental confirmations of the exact magnetobiological effects of geomagnetic variations when the magnetic component of a previously recorded geomagnetic storm was reproduced in laboratory conditions [28,71].

**Table 1:** Examples of biological effects of magnetic storms.

No	Object (Species)	Estimated Parameter	Effect, %	*f*, Hz	TVMF Induction (b)	Duration	*n*	Refs.
1	Human Adults, healthy, living above 70° north latitude	Amplitude of fluctuations in melatonin concentration in saliva	−20%	10^−5^	>80 nT	year	20	[60]
2	Human Adults, healthy, males, 23.9 ± 5.5 years (laboratory simulation)	The rate of blood movement through the capillaries	+30%	~7 × 10^−5^	~150 nT	18–24 h	8	[28]
		Systolic pressure	-N/A	—	—	—	—	
		Heart rate variability: HF LF VLF	+25% +25% +25%	— — —	— — —	— — —	— — —	
3	Human Adults, healthy, 26.1 ± 5.5 years Body mass index 23.9 ± 3.9 kg/m^2^ Heart rate 80.4 ± 5.4 bmp Systolic and diastolic pressure 114.5 ± 9.1 and 72.0 ± 8.1 mmHg. (laboratory simulation)	Heart rate variability: LF (incline 9.6°) HF (horizontal position)	−20% +40%	~7 × 10^−5^ —	~150 nT —	5–24 h —	8 —	[58,71]
		Correlation between changes in parameters of the cardiovascular system (HRV and capillary blood flow velocity) and the characteristics of the TVMF (B_x_, B_y_)	<0.05	—	—	—	—	
4	Human Adults, healthy, women, 24–49 years	Length of the RR interval with increasing oscillations of MF induction	+50%	0.01–3 Hz	20 (2–90) nT	2 days	17	[114]
5	Human Adults, healthy, women, 24–49 years	Regression coefficients of HRV signals with Ap index: HF LF VLF	200% 200% 200%	0.002–3.5 Hz (resonant 7.83 and ~14, 20, 26, 33, 39, 45)	20 (2–90) nT — —	2 days — —	17 — —	[29]
		Ratio LF/HF	−50%	—	—	—	—	
		Regression coefficients of HRV with induction of GMF: HF LF VLF	400% 150% 200%	— — —	— — —	— — —	— — —	
6	Human Population of 263 cities, data of National Center for Health Statistics (NCHS), USA	Risk of death from diseases: General	+50%	0.002–3.5	2–60 nT	2 days	>44 220 000	[59]
		Stroke	+50%	—	—	—	—	
		Myocardial infarction	+100%	—	—	—	—	
		Other cardiovascular diseases	+40%	—	—	—	—	
7	Human Patients of Nizhnekolomsk hospital, Penza region, Russia	Risk of heart attack Stroke risk	+50% +50%	0.002–3.5 —	200 nT —	2 days —	927 и 942	[106]
8	Human Analysis of archival data, men, women	Suicide rate	+70%	0.002–3.5	300 nT	2 days	1487	[115]
9	Human Patients of the Hospital of Kaunas University of Medicine, Lithuania	Risk of developing myocardial infarction without changes in the ST fragment on the ECG	+39%	0.002–3.5	>71 nT	1 day	2008	[68]
		Risk of developing myocardial infarction with changes in the ST fragment on the ECG	+54%	0.002–3.5	>71 nT	2 days		
10	Human Healthy volunteers of both sexes, 34–52 years old	Correlations (log(ρ)) of microcirculation oscillations with advising frequencies during geomagnetic disturbances ^1^: Endothelial Neurogenic Myogenic Respiratory Cardiac rhythm	2.0 2.0 2.5 1.0 0.5	0.01 0.03 0.1 0.3 1.0	>50 nT — — — —	2 days — — — —	9 — — — —	[62]
11	Human Men, women, age 25–65+ years, patients of Kaunas city hospital (geomagnetic latitude 52.38 N)	Risk of acute myocardial infarction	+10%	0.0016–5	>140 nT	1–4 days	13,629	[108]
		Risk of myocardial infarction	+63%	—	—	3 h	10,000	[107]
12	Human Men and women with myocardial infarction	Correlation between GMF induction and the risk of myocardial infarction (Women) ^1^	−0.5 −0.5 −0.5 N/A	3.5 7 15 32	>80 nT — — —	1 day — — —	435 — — —	[61]
		Correlation between GMF induction and the risk of myocardial infarction (Men)	−0.35 −0.35 −0.35 −0.35	3.5 7 15 32	— — — —	— — — —	268 — — —	
13	Human Men and women, 21–85 years	Systolic blood pressure, Diastolic blood pressure Average daily heart rate	+10% +10% +10%	0.0016–5 — —	>120 nT — —	24 h — —	447 — —	[98]
14	Human Men and women, 21–35 years (simulation in the laboratory)	Systolic blood pressure	+5%	0.0016	50 nT	24 h	3	[78]
		Heart rate	−5%	—	—	—	—	
		Heart rate variability: ULF (0.001–0.003 Hz) VLF (0.003–0.04 Hz) LF (0.04–0.15 Hz) HF (0.15–0.4 Hz)	+15% −10% −25% −25% −10%	— — — — —	— — — — —	— — — — —	— — — — —	
15	Human Pregnant women (healthy and pregnancy hypertension)	Risk of developing hypertension during pregnancy	+40%	0.0016–5	>200 nT	4 days	19,843	[109]
16	Human Men and women	Risk of ventricular tachycardia	−60%	0.0016–5	>120 nT	24 h	233	[109]
17	Human Men and women	Paroxysmal atrial fibrillation	−45%	0.0016–5	>130 nT	24 h	653	[116]
18	Human Men and women	Growth hormone Prolactin	+20% +30%	0.0016–5 —	>70 nT —	24 h	1752	[86]
19	Human Men and women, patients with atherosclerosis and healthy volunteers	Blood cholesterol concentration in atherosclerosis Triglyceride concentration in the blood of healthy people	−5% −7%	0.0016–5 —	>120 nT —	24 h —	1200 —	[85]
20	Human Men and women	Platelet count	+7% +5%	0.0016–5 —	>41 >70 nT	48 h —	1053 —	[82]
21	Human Men and women	Prothrombin time	+4% +8%	0.0016–5 —	>41 >70 nT	48 h —	1331 —	[83]
22	Human Men and women	ADP platelet aggregation	+25%	0.0016–5	>41 nT	24 h	162	[83]
23	Human Men and women	Fibrinogen concentration in blood	+11%	0.0016–5	>110 nT	24 h	100	[84]
24	Human Men and women	Average capillary closure time	+7%	0.0016–5	70 nT	24 h	120	[84]
25	Human Men and women	Basophils count Leucocyte count	−60% −40%	0.0016–5 —	70–120 nT —	24 h —	400 —	[67]
26	Human Men and women with migraine	Frequency of severe and moderate migraine episodes	+10% +32% +68%	0.0016–5 — —	40 70 120 nT	2 day — —	486 — —	[66]
27	Human Healthy ~41 years	Heart rate	−4%	0.0016–5	69 nT	24 h	14	[99]
		Heart rate variability (LF/HF ratio)	−15%	—	—	—	—	
		Well-being (survey)	−30%	—	—	48 h	—	
28	Human Men and women (21–35 years old)	Systolic pressure	+5%	0.0016	50 nT	24 h	3	[78]
		Heart rate	−5%	—	—	—	—	
		Heart rate variability: ULF (0.001–0.003) VLF (0.003–0.04) LF (0.04–0.15) HF (0.15–0.4)	+15% −10% −25% −25%	— — — —	— — — —	— — — —	— — — —	
29	Human Men and women (24–73 years old)	Systolic blood pressure relative value Sensitive people proportion	3% −32%	7.5–8.5 —	>1.97 pT —	24 h —	112 —	[117]
		Diastolic blood pressure relative value, sensitive people proportion	−3% −27%	— —	— —	— —	— —	
		Mean arterial pressure, relative value, Sensitive people proportion	−2% −30%	— —	— —	— —	— —	
		Heart rate	N/A	—	—	—	—	
		Depression score relative value Sensitive people proportion	−3% −20%	— —	— —	— —	— —	

^1^—Absolute values of correlation coefficients rather than effect sizes in % are shown in No. 12 and 15 (as in the original studies). These values have not been included in analyses of dependence of quantitative characteristics of biological effects of ELF-MFS on their frequency, induction, and duration (see below). Symbol “—” means that the value is the same as the previous one.

## 3. Magnetobiological Effects of Anthropogenic ELF-MFs

To simplify the description of the effects of ELF-MFs, we use a short notation of the spectral content: *f*(x)b(y1)B(y2)t(z), where b is the amplitude TLVF oscillations in μT, B is the amplitude of static MF (SMF) in μT, and *f* is the frequency in Hz, t—total exposure duration in units provided by the authors of the relevant works. Magnetobiological effects were conditionally divided into effects at the whole organism and cellular levels.

### 3.1. Effects on the Whole Organism (Laboratory Studies)

Much of the work shows that the main targets of ELF-MFs are the cardiovascular and nervous systems. [118]. The effects of ELF-MFs on the immune, musculoskeletal, and other systems have also been described [119].

**Figure 3:**
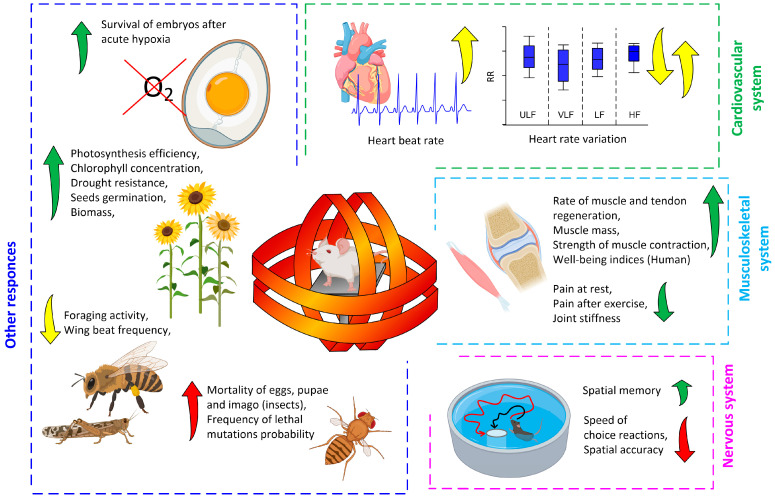
Non-thermal effects of ELF-MFs on an organism and its organ systems were discovered in laboratory conditions. The directions of the arrows indicate the direction of the effect: up—increasing the parameter, down—decreasing the parameter. The color indicates the expected impact of the effect on the organism being studied: green—positive, yellow—difficult to assess, red—negative. Source references are presented in the text and Table 1.

The beneficial effects of ELF-MFs on the musculoskeletal system were demonstrated in a rat tendon rupture model. The field *f*(40)b(1500)B(35)t(48 h) increased the force of contraction of the leg muscles in both operated and healthy animals, increased the surface area of the muscle, and accelerated the recovery of the force of contraction of the muscles of the operated limb [120] (Figure 3). The ELF-MF with the characteristics *f*(450)b(3500)B(38)t(200 min) has a positive effect on the functioning of joints and can be used in the treatment of pathologies of the musculoskeletal system, in particular, osteoarthritis [121].

An experimental ELF-MF *f*(16)b(28.3)B(39)t(18 h) reduced heart rate, total heart rate variability, and the power of low-frequency HRV oscillations in healthy volunteers during sleep [81]. These data are in agreement with the data obtained from studying the effects of magnetic storms (see above). Another study showed an increase in the power of low-frequency HRV components *f*(50)b(28)B(0.01)t(15 min) [122]. In this case, the differences between the results are explained by the use of a frequency of 50 Hz, near-zero static MF, and a shorter magnetic exposure time compared to most studies [29,78,81].

It is worth noting the effects of ELF-MFs on the functioning of cells of the immune system. In particular, it has been shown that the ELF-MF of complex form *f*(320 + 780 + 880 + 2600)b(5)B(50)t(30 min) has a mild anti-inflammatory effect, reducing the granularity of peripheral blood neutrophils in patients with previous coronavirus infection [123]. However, these data should be treated with caution since simple forms of the ELF-MFs *f*(7,8)b(24)B(4.1)t(72 h) or *f*(50)b(1000)B(0.001)t(48 h) reduce the viability of human cord blood lymphocytes [124,125]. ELF-MFs of complex shape, for example, *f*(1 + 4.4 + 16.5)b(600 + 100 + 160)B(42)t(1 h) or *f*(12.6 + 48.5)b(100)B(60)t(1 h), enhance fMLF-induced ROS generation by peripheral blood neutrophils [126,127].

ELF-MFs of the complex form *f*(6 frequencies from 5.1 to 6.98)b(100)B(60)t(28 h) increased tumor-induced secretion of proinflammatory cytokines TNF-α and IFN-γ by macrophages and T-lymphocytes in mouse blood by 2–3 times [128].

ELF-MFs influence the behavior of humans, other mammals, and invertebrates (e.g., insects) [129]. At the same time, both positive and negative effects of ELF-MFs on memory and learning have been described [119]. ELF-MFs also influence the search behavior of honey bees as well as the flight activity of desert locusts [130,131,132]. The ELF-MF application influences the spatial orientation of ants [133]. The influence of ELF-MFs on mammalian behavior can be explained by the activation of neurohumoral pathways, in particular the hypothalamic–pituitary–adrenal axis [134].

ELF-MFs affect a human’s spatial perception in selection and angle alignment tests. In this case, the effect of the ELF-MF is observed in a wide range of spectral content and duration *f*(20–120)b(12–98)B(0.01–50)t(1.5–5 h) [132,135].

The ELF-MF *f*(50)b(1000)B(0.001)t(10 h) causes an increase in Ca^2+^ concentration in the brain tissues of rodents. At the same time, different parts of the brain have different sensitivities to ELF-MFs. The cortex is the least sensitive, and the hippocampus is the most sensitive [136]. An ELF-MF with high induction blocks electrically excited postsynaptic potentials of hippocampal neurons *f*(15–100)b(500–100,000)B(45)t(20 min) [137]. The biomagnetic effect, in this case, depends to a greater extent on the field frequency (maximum at 15 Hz) and a lesser extent on induction (higher for 2–3 mT) [138,139,140]. On the contrary, an ELF-MF with low induction *f*(50)b(100)B(0.001)t(30 min) increases the amplitude and speed of electrical responses of hippocampal neurons to electrical stimulation [141]. In some cases, a series of stimulations with an ELF-MF causes a cumulative effect, even when the time of exposure to the field and resting is equal. Preliminary magnetic exposure has a more pronounced effect than magnetic exposure during or after electrical stimulation [142].

The blocking of ionotropic and metabotropic glutamate receptors (NMDAR and AMPA/kainate receptor) and calcium channels protects neurons from the blocking effect of ELF-MFs [136,139]. Everything points to a receptor-mediated action of ELF-MFs [139,142]. Since the target of ELF-MFs can be neurons, many authors have suggested that ELF-MFs can be used for the treatment of neurodegenerative diseases. In particular, the ELF-MF *f*(1)B(500)t(6000 s) protected neurons from apoptosis and improved the results of completing the Maurice water maze in mice with a model of vascular dementia [143].

The ELF-MF of a complex form *f*(0.38 + 4.88)b(80)B(42)t(40 h) improves spatial memory in mice with a model of familial and sporadic forms of Alzheimer’s disease and also inhibits the formation of amyloid plaques in hippocampal neurons [144].

A significant number works on plants related to SMF with inductions from 4 to 500 mT [145,146,147]. Works on ELF-MF variables are not numerous.

The ELF-MF *f*(12–33)b(1.3–5.4)B(42)t(24 h) causes a deviation in the angle of gravitropism in flax seedlings [55,148]. PeMFs can affect the mobility of unicellular algae; in particular, the ELF-MF *f*(16–18)b(20.9)B(52)t(48 h) significantly increases the mobility of diatoms in aqueous solution [149]. The effect largely depends on the concentration of Ca^2+^ in the solution and manifests itself at one of the cyclotron frequencies of calcium [149]. Seed treatment by the ELF-MF *f*(14.3–16.6)b(18–20)B(45–52)t(12 days) increases total plant biomass, the number and area of leaves, chlorophyll content, and photosynthesis efficiency [150,151,152]. The ELF-MF *f*(14.3)b(18)B(52)t(2 h) significantly reduces moisture loss by wheat seedlings during simulated drought and maintains photosynthetic efficiency and growth rates [151].

Depending on the spectral content the ELF-MF *f*(13–60)b(0.7–74)B(41)t(1–3 days) causes either acceleration or deceleration of planarian regeneration. The dependence is complex with the presence of amplitude–frequency “windows” in which the effect manifests itself [54,148]. TVMFs can disrupt embryogenesis in invertebrates [153]. PMPs reduce the survival rate of honey bees and slow down their development [154].

The ELF-MF *f*(60)b(2–10)B(40–50)t(20 min) even with a short exposure protects chicken embryos from the effects of acute hypoxia (1 h), increasing their viability after hypoxia by 3 times compared to untreated samples [155]. The protective effect of the ELF-MF against lethal hypoxia depended on the direction of the field and was 1.4 times higher for horizontal EMFs compared to vertical EMFs at 4 μT TVMF induction [155]. A significant part of the work on the effects of ELF-MFs on gametogenesis (oviposition) and embryonic development was carried out on the fruit fly *Drosophila melanogaster*. This is due to the convenience of research and the short life cycle of *D. melanogaster* [156,157]. A decrease in the number of eggs in the clutch, and hence suppression of gametogenesis, was found after ELF-MF exposure [155]. MFs with high induction *f*(50)b(2000–5000)B(50)t(48 h) affect survival [156]. Moreover, the effect depends on the development stage. ELF-MFs reduced the viability of embryos and pupae but increased the viability of larvae and imago [156].

The biological effects of TVMFs can occur within several generations after exposure. F1 offspring showed an increase in fertility and survival of adult individuals after exposure of parent flies to the *f*(50)b(2000)B(50)t(3 h) field. These characteristics were reduced for F2 and F3 at the same time. The ELF-MF *f*(50)b(2000)B(50)t(72 h) significantly (1.6–4.8 times) increased embryo mortality [158]. Long-term exposure to the ELF-MF *f*(50)b(500)B(50)t(500 days) accelerated the accumulation of recessive lethal mutations over 40 fruit fly generations [157]. The potential mechanisms of ELF-MF action on embryo survival are increased DNA fragmentation (field *f*(50)b(200)B(40)t(48 h)) [159] and/or increased expression of apoptosis inducers caspase-3 and caspase-9 [160]. It is noteworthy, that *D. melanogaster* embryos’ survival rate increases after low induction ELF-MF exposure *f*(50)b(5–40)B(0.2)t(3 h) [161].

### 3.2. Effects at the Molecular–Cellular Level (Laboratory Studies)

**Figure 4:**
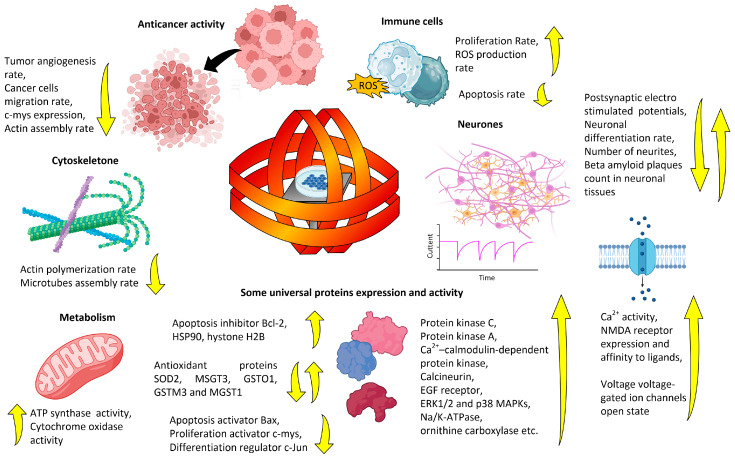
Non-thermal effects of ELF-MFs detected in laboratory conditions at the molecular–cellular level. The directions of the arrows indicate the direction of the effect: up—increasing the parameter, down—decreasing the parameter. References are presented in the text and Table 2.

ELF-MFs influence survival, proliferation, and DNA repair in normal human peripheral blood lymphocytes (Figure 4). ELF-MFs reduce the proportion of cells in apoptosis and accelerate their proliferation [162]. The biomagnetic effect depends on induction. The ELF-MF *f*(50)b(800)B(40)t(44 h) accelerated proliferation more strongly, did not increase the proportion of cells in apoptosis, and increased the number of cells containing micronuclei. The ELF-MF *f*(50)b(80)B(40)t(44 h) increased the proportion of cells with micronuclei and did not accelerate cell proliferation [162]. There is evidence of the influence of TVMFs on the redox potential of cells [163].

The ELF-MF *f*(16–315)b(1.75–61)B(38)t(15 min) enhanced the release of calcium Ca^2+^ by neurons in the chicken brain [164,165,166]. The effect slightly depends on the ELF-MF frequency [166,167]. At the same time, a field with similar characteristics, but a longer duration *f*(45)b(7–25)B(36.6)t(23 h) reduced the neuronal differentiation of PC-12 cells, which was expressed as a decrease in the number of cells with processes (neurites) and a decrease in the length of neurons [168,169,170]. This effect was confirmed by a double-blind method [170]. The ELF-MF *f*(16.3)b(40)B(20)t(30 min) significantly increased the calcium activity of rat bone marrow cells [171].

The ability of ELF-MFs to influence cell differentiation was described in many studies. The biomagnetic effect of ELF-MFs on neuronal differentiation depended on the direction of the field. A vertical ELF-MF *f*(45)b(30)B(36.6)t(23 h) decreased the proportion of differentiated cells (−60%), but a horizontal ELF-MF increased the proportion (+20%). The combination of fields gave an “intermediate” result. It reduced the proportion of differentiated cells but not so insignificantly (−30%) [169]. The effects of ELF-MFs on cell differentiation are highly dependent on cell type. For example, the ELF-MF *f*(1–50)b(100–300)B(4.1)t(7–35 days) did not affect the differentiation of human pluripotent immune cells into either granulocytic or lymphocytic types [172,173]. The effect on differentiation may depend on the magnitude of induction of the permanent ELF-MF component [169,172,173]. The ELF-MF *f*(50)b(0.4)B(18.5)t(30 min) influenced intracellular signaling by accelerating the clusterization of the epidermal growth factor receptor (EGFR) like a ligand and triggering the Ras small G-protein signaling cascade [174]. The effect depended on the signal shape. The sinusoidal field had a significant effect on the activation of the EGFR-dependent signaling pathway. Adding noise to a “pure” sinusoidal signal significantly inhibited this ELF-MF’s biomagnetic effect [174].

It has been shown on ion channel transfected cells, differentiated neurons, and hippocampal slice neurons that the ELF-MF *f*(15–60)b(500–2000)B(45–50)t(>1 min) can be targeted by VGICs [175,176,177]. Experimental evidence for the involvement of voltage-gated ion channels (VGICs) in the implementation of the magnetobiological effects of ELF-MFs has been described [178]. In general, L-type voltage-gated calcium channels act as ELF-MF targets [179]. T-type voltage-gated calcium-channel-dependent anticancer activity was also described [180]. Specifically, *f*(60)b(700)B(50)t(28 h) increased the proportion of chromaffin^+^ cells with neuronal morphology, neurite length, Ca^2+^ current, and KCl-evoked catecholamine release by neuronal cells [181]. ELF-MFs with a higher induction *f*(50)b(2000)B(44)t(48 h) increased Ca^2+^ influx, decreased intracellular pH, and increased the proportion of cells with neuronal differentiation (neurofilament^+^ and synaptophysin^+^ cells) and high expression Ca(v)1.2 and Ca(v)1.3 [175,182]. The effects of ELF-MFs may be mediated by changes in the activity of transcription factors, in particular CREB phosphorylation [175]. The TVMF *f*(15 or 50)b(500–2000)B(50)t(10–30 min) significantly modified the I-V curves for sodium and potassium VGIC change due to changes in the membrane potential at half activation/inactivation and the slope factor (activation/inactivation rate) of the VGICs in hippocampal slice neurons [177]. MF effects were blocked by L-type Ca^2+^ channels blocked by nifedipine or ω-conotoxin and enhanced by the L-type Ca^2+^ channel agonist Bay K-8644 [181,182]. The latter fact is one of the experimental pieces of evidence of VGIC’s participation in cell responses to TVMFs and realization of ion forced-oscillation mechanisms (see below) in biological systems.

Anti- and pro-tumorigenic effects of ELF-MFs are described in the literature. An ELF-MF with complex shapes *f*(5.1–6.98)b(100)B(60)t(28 h) significantly (more than 2 times) reduced tumor size when injecting Ehrlich ascitic carcinoma into mice and increased the survival of mice several times [128], which makes the use of ELF-MFs with complex shapes a potential approach to cancer therapy. The ELF-MF *f*(60)b(2000)B(50)t(3 h) disrupts the division processes of human neuroblastoma SH-SY5Y cells by disrupting the assembly of actin filaments and microtubules [183]. ELF-MFs affect chromatin conformation, determined by abnormal DNA viscosity [30]. ELFs with a frequency of 50 and 60 Hz induce stress responses in cells of the human promyelocytic lineage HL-60 [183].

The pro-oncogenic effect of ELF-MFs is supported by the data that a field with long-term exposure *f*(60)b(0.2)B(0.001)t(7 days) protected MCF-7 human breast carcinoma cells from the inhibitory effect of melatonin on their proliferation [184]. An ELF-MF with a high amplitude of GMF induction fluctuations *f*(50)b(500)B(35)t(30 min) also did not affect the viability of MCF-7 cells [185].

The effects of ELF-MFs at the molecular level include the following examples. An ELF-MF with high induction *f*(50)b(2000)B(40)t(4 days) decreased expression of the c-Jun protein (regulator of neuronal differentiation) in mice [186].

An ELF-MF of short duration *f*(9–18)b(21–30)B(43)t(15 min) is sufficient to change the chromatin conformation in both prokaryotes and eukaryotes. It is noteworthy that the effects of ELF-MFs depend on the organization of the genetic apparatus. Under the same magnetic conditions, chromatin unfolds in prokaryotes (an increase in AVTD), while in eukaryotes it condenses (a decrease in AVTD) [187,188].

The ELF-MF *f*(9–18)b(250–500)B(37)t(5–30 min) significantly changed the expression of antioxidant defense protein genes including superoxide dismutase, GSTO1, GSTM3, and MGST1 [189]. The effects of ELF-MF depended on both the maximum induction and the duration of magnetic exposure.

ELF-MFs affect the activities of enzymes involved in active ion transport (ATPase) and oxidative phosphorylation (cytochrome) [190,191]. The ELF-MF *f*(60)b(2–10)B(0.1)t (8–15 min) increased the activities of rabbit Na/K-ATPase and rat cytochrome oxidase. ELF-MFs with similar spectral content and duration increased the activity of ornithine carboxylase [192].

The ELF-MF *f*(60)b(8)B(0.1)t(20 min) enhances the expression of stress proteins in the cell, in particular the heat shock protein HSP70 [193]. ELF-MFs can enhance the expression of regulators of genetic expression and proliferation, including histone H2B and c-myc [194].

ELF-MFs may control circadian rhythms due to the disruption of melatonin production. In particular, it has been shown that *f*(50)b(100–250)B(1–26)t(1–7 days) causes a decrease in the concentration of melatonin in the blood plasma and pineal gland and an increase in the effect of the melatonin leader (6-sulfatoxymelatonin) in the urine [195,196,197]. The ELF-MF-induced *f*(50)b(0.01)B(49)t(80 days) change in melatonin synthesis in cows was season-dependent and more pronounced in winter time [198]. An ELF-MF with high induction *f*(50)b(1000)B(38)t(1 h) inhibits the activity of serotonin synthesis [199].

Proteomic analysis indicates that the high-induction ELF-MF *f*(60)b(2000)B(38)t(3 h) leads to alterations in the expression levels of 12% of all cell proteins. Among these, 7% exhibit an increase in expression, whereas 5% display a reduction in expression [200]. Among the target proteins of ELF-MFs, structural (actin), regulatory (kinases), participants in cell energy supply (ATPases and ATP synthase), histones, and others were found [200,201]. In addition to changes in expression, structural rearrangements are also detected: disruption of the integrity of actin filaments and microtubules [200]. An ELF-MF of complex form *f*(1 + 4.4 + 16.5)b(600 + 100 + 160)B(42)t(1 h) increases the rate of lipid peroxidation in mouse whole blood neutrophils [126].

ELF-MFs change the permeability of bilipid membranes for Ca^2+^, and the effect depends on MF spectral content. Membrane permeability increased in the case of *f*(25.5)b(31)B(37)t(1 h) and decreased in the case of *f*(20)b(37)B(37)t(1 h) [202]. ELF-MFs, under some conditions, can cause oxidative damage to the DNA molecule, measured by the generation of 8-oxoguanine [203]. 

The ELF-MF *f*(60)b(1500)B(0.47)t(144 h) inhibited cell proliferation via G1 phase arrest and activation of the ATM-Chk2-p21 pathway [204]. The ELF-MF *f*(50)b(7–1000)B(0.01)t(15 min) increased phosphorylation of ERK1/2 and p38 MAPK, but not JNK [205]. The degree of phosphorylation was determined by TVMF induction and magnetic exposure time. The ELF-MF *f*(16.3)b(40)B(20)t(30 min) significantly increased the calcium activity of rat bone marrow cells [171]. ELF-MFs also increased the activities of protein kinases C and A, Ca^2+^-calmodulin-dependent protein kinase, calcineurin, and the affinity of the NMDAR receptor for glutamine *f*(50)b(100)B(39)t (90 days) [206].

The ELF-MF *f*(50)b(400)B(45)t(6 h-26 days) increases the activity of the RKIP-dependent signaling pathway and activation of the transcription factor NF-κB in control rats and animals with simulated Alzheimer’s disease. Specifically, ELF-MF administration improved behavioral test scores and restored normal intracellular signaling. [207].

The ELF-MF *f*(50)b(1000)B(60)t(1 h) caused increased generation of ROS (singlet oxygen, superoxide, hydroxyl radical, and peroxides), decreased activity of antioxidant systems, and the development of oxidative stress and epigenetic changes have also been shown in neuronal cells [208,209]. The ELF-MF *f*(50)b(1000)b(60)t(>6 h) altered the expression of more than 90 mitochondrial and oxidative stress genes, including neuronal nitric oxide synthase, neuropeptide FF receptor 1, alpha-synuclein, and neuronal tropomodulin 2 [208]. In general, the circuit of a signal stage can be represented as follows: antioxidant system activity↓ ↔ ROS production↑ → protein carboxylation↑ → mitochondria and pro-oxidant genes↑ → lipid peroxidation, DNA-RNA damage, etc.↑ [208,210].

Despite the diversity of effects, an ELF-MF’s action can be generally represented as a sequence of “cellular stress response”: disruption of protein self-assembly or/and ROS production → cell cycle arrest → SHP and other chaperone activation and chromatin reparation activation → activation of NF-κB-, MAPK-dependent and other signaling pathways → removal of damaged molecule (via the ubiquitin-dependent pathway) or cell death via apoptosis [134,211]. The molecular and cellular mechanisms of these processes are described in more detail in the reviews [134,210].

ELF-MFs can influence the rates of self-organizing reactions outside living systems. For example, the ELF-MF *f*(60)b(28)B(0.1)t(20 min) increased the frequency of oscillations of the redox potential of the Fe^2+^/Fe^3+^ pair in a Belousov–Zhabotinsky reaction [212]. It is noteworthy that the frequency of the ELF-MF was significantly (~2000 times) higher than the frequency of the Belousov–Zhabotinsky reaction (~0.03 Hz). The effects of ELF-MFs have also been demonstrated in other model reactions [213]. The effects TVMFs can also manifest themselves at the level of water and aqueous solutions of proteins [214,215]. In particular, a TVMF with a frequency of 50 Hz and an induction of 50 μT causes an increase in the spontaneous chemiluminescence of aqueous solutions of immunoglobulins [216]. There is evidence in the literature about the ability of weak EMFs to influence the redistribution of charges in protein molecules and, as a consequence, change their conformation [217]. The activity of alpha-amylase immobilized on supermagnetic particles depends on the frequency of the rotating MF of 5–10 Hz [218]. More detailed information on magnetobiological effects of ELF-MF is given in Table 2.

**Table 2:** Examples of biological effects of anthropogenic and laboratory-generated ELF-MFs.

No	Object (Species)	Characteristics	Effect, %	*f*, Hz	Induction		Duration	*n*	Statistic	Installation Type	Installation Size	Verification	JSR	Refs.
					b (TVMF)	B (SMF)								
1	Human cord blood lymphocytes	Viability	−15% −20% −26%	7.8 — —	6, 17, 24 μT	4.1 µT — —	72 h — —	6 — —	One-way ANOVA, post hoc Fisher LSD	System of perpendicular coils (2 axes)	10 × 10 cm	Magnetometer, 3D-map variation <5% The external field was reduced by a μ-metal chamber to 3.7 μT.	0.42	[124]
2	Human pluripotent cell line iPS (7F3955- pMXs#1)	Proportion of CD34 + CD38—cells (differentiated)	N/A N/A N/A N/A	50 — — —	0 100 200 300 mT	4.1 µT — — —	7 day — — —	5 — — —	One-way ANOVA, post hoc Fisher LSD	Helmholtz coils (1 axis)	Ø 20 cm	Magnetometer, one point, variation <5%. The external field was reduced by a μ-metal chamber to 3.7 μT.	0.42	[172]
3	Fire ants *Solenopsis* sp. Imago	Time to escape the trap	−20% +30% −50%	60 — —	21 40 57	26 29 26	200 s — —	30 — —	Rayleigh test, Watson U2 test	Helmholtz coils (1 axis)	18 × 18 cm	Magnetometer, time profile of ELF-MF was shown GMF 21 μT	0.3	[133]
		Proportion of insects moving along the line GMF	−8% −8%	— —	57 40 μT	10 26 μT	— —	— —						
4	Planaria *Girardia tigrina* Asexual laboratory race, length 7–8 mm	Regeneration index (amputation of 1/5 body part)	+20% +30% +15% +2% +28% +2% +12% +0% +11% −18%	60 — — — — — — — — —	29 55 88 105 164 227 265 311 361 412	42 μT — — — — — — — — —	3 days — — — — — — — — —	30 — — — — — — — — —	Student’s *t*-test	Helmholtz coils	Ø 30 cm	Magnetometer, one point, TVMF ambient 50 Hz 5 nT	0.18	[148]
	Flax *Linum bienne* upper segments of stems without leaves 2.5 cm long	Deviation of the apical end of a segment from the horizontal plane (gravitropism)	+3.5% +2% +3% +2%	— — — —	55 105 164 227 μT	— — — —	2 h — — —	20 — — —						
5	Planaria *Schmidtea mediterranea*, Asexual laboratory race, length 10 mm	Rate of growth of the planarian head blastema	−10% −24% +3% +25% +5%	13 16 27 30 33	74 μT — — — —	41 μT — — — —	24 h — — — —	5 — — — —	ANOVA	Helmholtz coils	Ø 30 cm	Magnetometer, one point, TVMF ambient 50 Hz <6 nT	0.79	[54]
6	Cows *Bos taurus* Males and females, adults	Orientation in space in the north-south direction (Satellite observation, image analysis)	−99%	50- 60	5–15 μT	~40 μT ^1^	24 h	1699	Rayleigh test, Watson–Williams F test, Mardia–Watson–Wheeler test	High-voltage power lines	50 × 150 cm	Not applicable	4.03	[64]
	Roe deer *Capreolus capreolus* Males and females, adults		−99%	—	—	—	—	653						
7	Honey bee *Apis cerana* Larvae (2 days)	Survival	−60%	50	3 mT	~50 µT	20 days	72	Duncan’s post hoc test, Dunnett’s post hoc test, Log-rank (Mantel–Cox) test	Commercially available ELF-EMF generator (Litian magnetic and electric Science and Technology Co., Ltd., Mianyang, China)	15 × 10 × 10 cm	ELF-FM ≫ GMF	0.68	[154]
		Body mass	−10%	—	—	—	—	—						
		Duration of development	+5%	—	—	—	—	—						
		Gene expression: increasing	+153 genes	—	—	—	—	—						
		decreasing	−269 genes	—	—	—	—	—						
8	Human Adults, healthy, 26.1 ± 5.5 years, body mass index 23.9 ± 3.9 kg/m^2^, heart rate 80.4 ± 5.4 beats/min Systolic pressure 114.5 ± 9.1 mmHg Diastolic pressure 72.0 ± 8.1 mmHg.	The rate of blood movement through the capillaries	+30%	7 × 10^−5^	205 nT —	49 µT —	18–24 h —	8 —	F test (CBV and BP), factorial ANOVA (RR intervals)	Helmholtz coils (3 axes) imitation of a magnetic storm k = 7	2.5 × 2.5 × 2.5 m	Magnetometer, one point, variation <0.03%. Noise and GMF were compensated	0.65	[28,71]
		Systolic pressure	N/A	—	—	—	—	—						
		Heart rate variability: HF LF VLF	+25% +25% +25%	— — —	— — —	— — —	— — —	— — —						
		Correlation between changes in parameters of the cardiovascular system (HRV, capillary blood flow velocity) and characteristics of TVMF (Bx, By)	<0.05	—	—	—	—	—						
9	Human Adults, healthy, 26.1 ± 5.5 years, body mass index 23.9 ± 3.9 kg/m^2^	Heart rate variability: LF (tilt 9.6°) HF (horizontal position)	−20% +40%	7 × 10^−5^	205 nT —	49 µT —	5–24 h —	8 —	Factorial ANOVA	Helmholtz coils (3 axes) imitation of a magnetic storm k = 7 and microgravity	2.5 × 2.5 × 2.5 m	Magnetometer, one point, variation <0.03%. Noise and GMF were compensated	1.03	[58]
10	Human leukemia cells K562	HSP70 protein concentration	+100% +50%	50 —	25 100 μT	41.8 μT —	1 h —	3 —	Non-parametric Chi-square test, Kruskal–Wallis test, ANOVA, Dunnett’s post hoc test	Helmholtz coils	-	Magnetometer, one point, variation <0.5 μT	0.45	[219]
11	Mice Males and females, 10 and 15 days, respectively	Protein expression: c-Jun c-Fos (markers of neuronal differentiation)	−15% N/A	50 —	2 mT —	40 μT —	4 days —	3 —	Student’s *t*-test	Solenoid	-	Temperature variation <0.1 °C	0.4	[186]
12	*Escherichia coli* strains K12 AB1157 K12 EMG2 K12 GE499 K12 GE500 Human lymphocytes (men, ~30 years old, non-smokers)	Chromatin conformation measured by anomalous viscosity time dependencies (AVTD):	+25% +20% +5% +30% N/A −20% −20% N/A N/A N/A N/A −10%	9 12 16 18 25 60 9 12 16 18 25 60	30 μT — — — — — — — — — — —	43 μT — — — — — — — — — — —	15 h — — — — — — — — — — —	8 — — — — — — — — — — —	Student’s *t*-test	Helmholtz coils	-	Magnetometer, one point, variation, temperature variation <0.1 °C, GMF 43 μT (collinear) 19 μT (perpendicular)	0.86	[187]
13	Human breast cancer cells MCF-7	Cell survival	N/A	50	500 μT	37 μT	30 min	8	ANOVA, Bonferroni post hoc test	Solenoids system	44 × 14 cm	Magnetometer, one point	0.4	[189]
		Expression of genes of the antioxidant system: SOD2	−40%	50	250 μT	—	30 min	—						
		MSGT3	+36% +20%	— —	— —	— —	15 min 5 min	— —						
		GSTO1	−40% −14% −23%	— — —	— — —	— — —	5 min 15 min 30 min	— — —						
		GSTM3	−31% −33% +33%	— — —	— — —	— — —	5 min 15 min 30 min	— — —						
		MGST1	+36% −37%	— —	— —	— —	30 min 15 min	— —						
14	*Gallus gallus* spp. *domesticus* chicks 5 days after hatching	Release of Ca^2+^ from brain tissue	+13%	315	61 nT	38 μT	20 min	32	One-way ANOVA	Helmholtz coils (1 axis)	Ø 47 cm	Magnetometer, one point, GMF ~38 μT	0.42	[166]
15	*Gallus gallus* spp. *domesticus* chicks 5 days after hatching	Release of Ca^2+^ from brain tissue	+11% +13% +14% +11% +18% +14% +15% +9% +14%	45 50 60 15 45 60 75 90 105	61 nT — — 61 nT — — — — —	38 μT	20 min — — 20 min — — — — —	32	Two-way ANOVA	Helmholtz coils (1 axis)	Ø 47 cm	Magnetometer, one point, GMF ~38 μT	0.42	[167]
16	Neuronal cell line PC-12	Neurite growth rate	−5% −25% −75% −75% −40% −20%	45 — — — — —	7.0 14, 20 25 37 46 μT	36.6 μT — — — — —	23 h — — — — —	3 — — — — —	Bessel function	Helmholtz coils (2 axes)	Ø 20 cm	Magnetometer, one point, variation SMF <0.2 μT. Ambient TVFM 60 Hz, <0.9 μT	0.42	[168]
17	Neuronal cell line PC-12	Percentage of cells with neurites	+20% −30% −60%	45 — —	20 ↔ 30 ↔↕ 30 ↕ μT	36.6 μT — —	23 h — —	3 — —	Student’s *t*-test	Helmholtz coils (2 axes)	Ø 20 cm	Magnetometer, one point, variation SMF <0.2 μT. Ambient AFM 60 Hz, <0.9 μT	0.79	[169]
18	Neuronal cell line PC-12	Percentage of cells with neurites (double-blind experiment)	−70%	45	23.8 μT	36.6 μT	23 h	3	Double-blind test, binomial test	Helmholtz coils (2 axes)	Ø 20 cm	TVMF 50 Hz <0.08 μT SMF <0.36 μT. The external field was reduced by a μ-metal chamber	0.42	[170]
19	*Gallus gallus* spp. *domesticus* chicks 5 days after hatching	Release of Ca^2+^ from brain tissue	+12% +13% +15% +14% +12% +11%	16 — — — — —	1.75 3.85 5.57 6.82 7.65 7.77 μT	<0.1 μT	20 min	32	Two-way ANOVA	Helmholtz coils (1 axis)	Ø 47 cm	Magnetometer, one point GMF 38 μT	0.42	[165]
20	Rabbit kidney Na/K-ATPase *Oryctolagus cuniculus domesticus*	Enzyme activity	+10%	60	310 нT	<0.1 μT	15 min	3	Enzyme kinetics analysis methods	Specially designed and verified installation	-	Magnetometer, 3D map, variation < 3% MF in the thermostat < 0.1 μT	0.72	[190]
21	Cytochrome oxidase, rat liver of *Rattus norvegicus* Sprague–Dawley	Enzyme activity	+5% +15% +20% +40%	60 — — —	2 5 7 10 мкT	<0.1 μT — — —	8 min — — —	3 — — —	Enzyme kinetics analysis methods	Specially designed and verified installation	-	Magnetometer, background MF < 0.1 μT	0.72	[191]
22	Fibroblast line L929	Ornithine carboxylase activity	+40% +80% +80% +110% +80% +100%	60 — — — — —	4 5 6 8 9 20 μT	0 μT — — — — —	4 h — — — — —	5–10 — — — — —	Two-tailed Student’s *t*-test	Helmholtz coils	Ø 10.5 cm	Magnetometer, one point, variation <15%	0.72	[192]
23	Belousov–Zhabotinski (BZ) reaction Starting frequency 0.03	Frequency of cycles of changes in the redox potential Fe^2+^/Fe^3+^ at a temperature of 15–19 °C	+5%	60	28 μT	0.1 μT	20 min	8	Regression analysis methods	Helmholtz coils	13 × 14 cm	Magnetometer, one point, SMF variation < 0.1 μT. GMF shielded with μ-metal	0.78	[212]
24	Hela cell line after heating 43 °C for 20 min	SHP70 expression	+15% +60%	60 —	8 80 μT	20 μT —	20 min —	3 —	Tukey test, normality Kolmogorov–Smirnov test	Solenoid	5.27 × 25.0 cm	Magnetometer, one point. GMF 20 μT	0.88	[220]
25	Endothelial cells: SPAE	Inducible (heating 44 °C 30 min) HSP70 protein level	N/A +46% +45% +71% +78% +79%	50 — — — — —	150 300 680 μT — — —	12 μT	24 h — 8 16 24 48	3	Student’s *t*-test	Solenoid	Not discribed	1–12 μT (without experiment) 2–16 μT (during experiments) Magnetometer, 3D map, accuracy < 2 μT	0.79	[221]
	HUVECs		+40%	—	—	—	24 h	—						
	Human leukemia and lymphoma cells: CEM		+60%	—	—	—	—	—						
	HL-60		+65%	—	—	—	—	—						
	U937		+61%	—	—	—	—	—						
26	Human promyelocytic lineage cells HL-60 (lymphoblasts)	Chloramphenicol acetyltransferase (CAT) activity	+150%	60	8 μT	<0.1 μT	20 min	3	Student’s *t*-test	Helmholtz coils (1 axis) in a μ-metal container	13 × 14 cm	Magnetometer, one point, SMF variation <0.1 μT. GMF shielded with μ-metal (90 times reduction)	0.78	[193]
		HSP70 mRNA expression	+80%	—	—	—	—	—						
		HSP70 protein concentration	+210%	—	—	—	—	—						
27	Chicken *Gallus gallus* spp. *domesticus* White Leghorn, fertilized eggs	Embryo survival after 1 h of hypoxia	N/A +100% +200% +200% N/A +50% +100% +150%	60 — — — — — — —	2↕ 4 8 10 μT 2↔ 4 8 10 μT	40–50 μT — — — — — — —	20 min — — — — — — —	40 — — — — — — —	*x*^2^ analysis	Helmholtz coils (1 axis)	Ø 2 m	Magnetometer, one point, SMF <0.5 μT. GMF 40–50 μT	0.72	[155]
28	Human breast cancer cell line MCF-7	Melatonin-induced proliferation inhibition 10^−9^ M	100% 100%	60 —	0.2 1.2 μT	0 μT	7 days —	5	ANOVA	Merritt’s coils (2 axis)	16 × 16 × 16 cm	Magnetometer, one point, variation, SMF <5%, GMF and 60 Hz, 1.4 μT, TVMF <2%	2.16	[184]
29	Children, boys and girls, healthy or with leukemia	Risk of developing leukemia	×1.27–3.13	50– 60	≥0.4 μT	~45 μT	>1 year	10,338 3203	*x*^2^ analysis	Meta-analysis of the assessment of the magnetic situation in cities	Not applicable	Not applicable	2.78	[70]
30	Children, boys and girls, healthy or with leukemia	Risk of developing leukemia	×1.2–2.13	50– 60	≥0.3 μT	35–45 μT	>1 year	meta-analysis	Inverse-variance weighted (Woolf), Mantel–Haenszel, and maximum-likelihood (ML) tabular methods, and using ML logistic regression	Meta-analysis of the assessment of the magnetic situation in cities	Not applicable	Not applicable	1.96	[69]
31	Chinese hamster lung cells (CHL)	Epidermal growth factor receptor (EGFR) clustering, qualitatively: sinusoidal field, sine + noise	++ +	50 —	400 μT —	18.5 μT —	30 min —	3	ANOVA and least significant difference (LSD) test	Helmholtz coils (3 axes)	Ø 36 cm	Magnetometer, oscilloscope SMF <18.5 μT TVMF 50 Hz, <1–2 μT	0.62	[174]
		Phosphorylation of signaling protein Ras: sinusoidal field sine + noise	+90% +5%	— —	— —	— —	— —	— —						
32	Diatom *Amphora coffeaeformis*	Mobility at a frequency of 16 Hz at different Ca^2+^ concentrations: 0.1 мM 0.25 мM 0.5 мM	+200% +900% +300%	16 16 16	20.9 μT — —	52 μT — —	2 days — —	12 — —	*x*^2^ analysis and ANOVA	Helmholtz coils (3 axes)	Ø 23 cm	Magnetometer, one point, variation <30 nT. GMF 52 μT TVMF ambient 60 Hz, <0.1 μT	0.42	[149]
		Mobility at Ca^2+^ concentration 0.25 mM and frequencies	+200% +500% +600% N/A	14 16 18 32	— — — —	— — — —	— — — —	— — — —						
33	Human bone marrow cell line TE-85	Ca^2+^ release	+120%	16.3	40 μT	20 μT	35 min	6	Student’s *t*-test	Helmholtz coils (3 axes)	Ø 30 cm	Magnetometer, one point. GMF 40 μT	0.97	[171]
34	Rats Wistar, males, adult	Concentration of 6-sulfatoxymelatonin in urine at night	+15%	50	100 μT	1 μT	24 h	5	Student’s *t*-test	Helmholtz coils (1 axis)	Ø 42 cm	Magnetometer, one point	0.42	[195]
35	Rats Wistar, males, adult	Serotonin-N-acetyltransferase activity	−10%	50	1 mT	38 μT	1 h	48	ANOVA followed by the Student–Newman–Keuls test	Solenoid (1 axis)	20 × 20 cm	Magnetometer, one point	0.4	[199]
36	Rats Wistar–King, males 11–18 weeks, 300–370 g.	Melatonin concentration at midnight in the pineal gland	20% −40%	50 —	5 250	26 μT —	6 weeks	400 —	Student’s *t*-test	Helmholtz coils	-	Magnetometer, one point, variation, TVMF 50 Hz <16 nT SMF <2% GMF 40 μT (total) 26 μT (horizontal)	0.42	[196]
		Melatonin concentration at midnight in the blood plasma	−20% −25%	— —	5 250 μT	— —	— —	— —						
37	Human Men and women (21–35 years old)	Systolic pressure	+5%	0.0016	50 nT	40 nT	24 h	3	Student’s *t*-test at a significance level of 0.001	Helmholtz coils (magnetic storm simulation)	3 × 3 × 3 m	Magnetometer, one point	1.37	[78]
		Heart rate	−5%	—	—	—	—	—						
		Heart rate variability ULF (0.001–0.003) VLF (0.003–0.04) LF (0.04–0.15) HF (0.15–0.4)	+15% −10% −25% −25% −10%	— — — — —	— — — — —	— — — — —	— — — — —	— — — — —						
38	Human Human peripheral blood lymphocytes	Proportion of apoptotic cells	−45% −36%	50 —	80 800 μT	40 μT —	44 h —	3 —	Two-way ANCOVA, and the Tukey honest significant difference (HSD) test	Helmholtz coils (1 axis)	42 cm Ø 20 cm	Magnetometer, one point, variation <1%	0.42	[162]
		Nuclear division index (NDI)	+5% +25%	— —	80 800 μT	— —	— —	— —						
		Proportion of cells with micronuclei	+15% −40%	— —	80 800 μT	— —	— —	— —						
39	Human neuroblastoma cell line SH-SY5Y	Survival cells	−15%	60	2 mT	38 μT	3 h	10	Student’s *t*-test for extremely low samples	Rodin’s star-coil	Ø 30 cm	Magnetometer, 3D map, ELF-MF≫ GMF	0.42	[200]
		Number of cells	−60%	—	—	—	—	—						
		Cell proteome analysis: increase in expression, decreased expression	+7% +5%	— —	— —	— —	— —	— —						
		Expression of individual proteins: prohibitin	+90%	—	—	—	—	—						
		4-HNE	−90%	—	—	—	—	—						
		F-actin	qualitatively	—	—	—	—	—						
		Guanine nucleotide-binding protein subunit beta-5,	+30%	—	—	—	—	—						
		Alpha-tubulin	+39%	—	—	—	—	—						
		Prohibitin	+13%	—	—	—	—	—						
		Alpha-ketoglutarate-dependent dioxygenase FTO	1/2.3	—	—	—	—	—						
		Serine/threonine-protein kinase 32C	×12.07	—	—	—	—	—						
		T-complex protein 1 subunit alpha	−41%	—	—	—	—	—						
		ATP synthase subunit beta, mitochondrial	+41%	—	—	—	—	—						
		Peptidyl-prolyl cis-trans isomerase FKBP4	+48%	—	—	—	—	—						
		PDZ and LIM domain protein 3	+72%	—	—	—	—	—						
		Sin3 histone deacetylase corepressor complex component SDS3	+31%	—	—	—	—	—						
		Nuclear fragmentation	+35%	—	—	—	—	—						
		Actin filament disruption	+35%	—	—	—	—	—						
		Disruption of β-tubulin strands	+35%	—	—	—	—	—						
40	Meta-analysis of articles on the relationship between the risk of developing amyotrophic lateral sclerosis Data from 62 articles. Women, >18 years old. USA, Denmark, Sweden, Switzerland	Development risk Mortality	+14%	50– 60	0.3–2.5 μT	~36 μT	>1 year	~20,000	Pooled analysis of the large sample size	Industrial fields	Not applicable	Not applicable	0.42	[65]
41	Human Men, healthy, 18–27 years old, body mass index 24 ± 2	Heart rate (HR) HR variability (HRV) VLF LF HF	−5% +10% +300% +200% +50%	50 — — — —	100 nT — — — —	28 µT — — — —	15 min — — — —	17 — — — —	ANOVA, corrected degrees of freedom via Greenhouse–Geisser estimates of sphericity if the assumption of sphericity was violated. *t*-tests with Bonferroni correction	Helmholtz coil (1 axis)	Ø 70 cm	Magnetometer, one point, variation SMF < 2 µT (26–30 µT), GMF 44 µT, TVMF 50 Hz 0.01 µT	0.42	[122]
42	People, men and women, 25.6 ± 4 years	Final angle of the line after adjustment SVV: standard deviation	−12% −12% −12% −12%	20 60 120 160	98 32.8 16.4 12.3	~50 μT — — —	1.5 h — — —	33 — — —	Eta squared (η_G_^2^) after ANOVAs	Single coil system (1 axis)	Ø 20 cm	Magnetometer, one point (dB/dt = 12.3 T/s)	0.42	[135]
		SVV	+10% +10% +10% +10%	20 60 120 160	98 32.8 16.4 12.3	— — — —	— — — —	— — — —						
		Angle setting time	−70% −70% −70% −70%	20 60 120 160	98 32.8 16.4 12.3 mT	— — — —	— — — —	— — — —						
43	Rats 200–250 g body mass, 3 months old, control and after tendon trimming surgery	Muscle mass: control, operated	+10% +25%	40 —	1.5 mT —	~30 μT —	45 h —	8 —	ANOVA, Tukey’s post hoc test	Helmholtz coils (1 axis)	Ø 60 cm	Magnetometer, one point	0.42	[120]
		Muscle surface area: control, operated	+2% +12%	— —	— —	— —	— —	— —						
		Strength of muscle contraction: control, operated	N/A +50%	— —	— —	— —	— —	— —						
		Time of maximum contraction: control, operated	N/A −10%	— —	— —	— —	— —	— —						
		Relaxation time at 80% (both)	N/A	—	—	—	—	—						
		Contraction force: operated	+60%	120	—	—	—	—						
44	Human Men and women after SARS-CoV-2 infection, age 50–70 years	Granularity of peripheral blood granulocytes	−10%	320+780+ 880+ 2600	5 μT	~50 μT	30 min	32	*t*-test after Shapiro–Wilk test	Ring-shaped portable generator	Ø 50 cm	Magnetometer, one point ELF-MF— GMF	0.42	[123]
		Peripheral blood granulocyte count	−10%	—	—	—	—	—						
45	Rats Sprague–Dawley, males, 14–18 days, hippocampal slices	Cell responses to electrical stimulation (normalized amplitude)	−25% −27% −30% −20% −22% −25% −8% −10% −15%	15 — — 50 — — 100 — —	0.5 1 2 0.5 1 2 0.5 1 2 mT	~45 μT — — — — — — — —	20 min — — — — — — — —	5 — — — — — — — —	ANOVA on Tukey’s multiple comparisons test	Solenoid (1 axis)	Ø 10 cm	Magnetometer, one point, variation SMF < 5% TVMF < 5% ELF-MF ≫ GMF	0.93	[138]
46	Rats Sprague–Dawley males, 14–18 days, hippocampal slices (CA1 region)	Electrically excited postsynaptic potentials	−30% −25% −20% −35% −25% −25% −35% −25% −25%	15 — — 50 — — 100 — —	0.5 1 2 0.5 1 2 0.5 1 2 mT	~45 μT — — — — — — — —	10 s — — — — — — — —	5 — — — — — — — —	Two-way ANOVA, Tukey’s multiple comparisons test	Commercially available systems XcELF (IT’IS Foundation, Zurich, Switzerland)	Not described	Magnetometer, one point, variation SMF < 5% TVMF < 5% ELF-MF ≫ GMF	0.79	[139]
47	Rats Sprague–Dawley, males, 14–18 days, hippocampal slices (CA1 region)	Electrical response to high-frequency electrical stimulation: in MF: control, against the background of receptor blockers NMDAR	−80% −40%	15 —	2 mT —	~45 μT —	20 min —	5 —	Two-way ANOVA, Tukey’s multiple comparisons test	Solenoid (1 axis)	Ø 10 cm	Magnetometer, one point, variation SMF < 5% TVMF < 5% ELF-MF≫ GMF	0.85	[139]
48	Rats Sprague–Dawley, males, 14–18 days, hippocampal slices (CA1 region)	Amplitude and slope of the electrical response to electrical stimulation (control): 20 min 40 min 60 min in the presence of AMPA/kainate receptor antagonist (10 μM CNQX)	−5% −20% −25% recovery after washin 100%	15 — — —	2 mT — — —	~45 μT — — —	20 40 60 min —	5 — — —	Two-way ANOVA on Tukey’s multiple comparisons test	Solenoid (1 axis)	Ø 10 cm	Magnetometer, one point, variation SMF < 5% AMF < 5% ELF-MF ≫ GMF	1.04	[142]
49	Rats Wistar embryos and newborns, slices of the hippocampus and neocortex	Electrical activity of neurons in response to electro-stimulation: Amplitude between minimum and maximum (bark) embryos, newborns	N/A +10% +15% +30% +45% +45% +50%	50 — — — — — —	0.5 1.75 2.0 2.25 2.5 2.75 3.0 mT	0.5 mT — — — — — —	7 days — — — — — —	7 — — — — — —	ANOVA or Student’s *t*-test	Helmholtz coils (1 axis)	Ø 42 cm	Magnetometer, one point, variation TVFM <25 μT, Variation SMF < 10 μT	0.64	[140]
		Maximum of response: embryos	+80% +100% +100% +100%	— — — —	2.25 2.5 2.75 3.0 mT	— — — —	— — — —	— — — —						
		Maximum of response: newborns	+80% +100% +100% +100%	— — — —	2.25 2.5 2.75 3.0 mT	— — — —	— — — —	— — — —						
		Action potential: embryos	+25%	—	2 mT	—	—	—						
50	Mice BALB/c, males, 12–13 weeks, 20–30 g	Ca^2+^ concentration in brain tissue: intact: bark cerebellum hippocampus brain stem	+10% +15% +350% +75%	50 — — —	1 mT — — —	<1 nT — — —	10 h — — —	8 — — —	One-way ANOVA, least significant difference (LSD) test	Helmholtz coils (1 axis)	Ø 40 cm	Magnetometer, one point GMF, magnetic force lines were parallel to the horizontal component of the local GMF	0.1	[136]
		Ca^2+^ concentration in brain tissue against the background of calcium channel blocker Amlodipine: bark cerebellum hippocampus brain stem	N/A +8% N/A N/A	— — — —	— — — —	— — — —	— — — —	— — — —						
51	Rats Wistar, males, 200 g, hippocampal neurons	Electrical response: first peak amplitude, second peak amplitude	+30% +20%	50 —	100 μT —	<1 μT —	180 h —	5 —	ANOVA Tukey’s test	Solenoid (1 axis)	Ø 20 cm	Magnetometer, one point	0.85	[141]
52	Rats Wistar, males, 21 days, hippocampus	Ca^2+^ concentration in cells	+200% +300%	50 —	50 100 μT	39 μT —	90 days —	3 —	Student’s *t*-test	Helmholtz coils (3 axes)	0.5 × 0.5 × 0.5 m	GMF vertical 15.89 ± 0.14 μT horizontal 39.43 ± 0.01 μT	0.8	[206]
		Enzyme activities: Protein kinase C	+15% +50%	— —	50 100 μT	— —	— —	— —						
		Protein kinase A	−55% −75%	— —	50 100 μT	— —	— —	— —						
		Ca^2+^–calmodulin-dependent protein kinase	+50% +75%	— —	50 100 μT	— —	— —	— —						
		Calcineurin specific activity	N/A N/A	— —	50 100 μT	— —	— —	— —						
		Phosphotases (total)	N/A	—	50 μT	— —	— —	— —						
		Ligand binding NMDAR (^3^H-L-glutamine)	−25%	—	100 μT	— —	— —	— —						
53	Children living in Mexico City: diagnosed with B-line acute lymphoblastic leukemia and healthy. Age in both groups 16 years	B-lineage acute lymphoblastic leukemia risks (case/control ratio)	+26% +53% +87% +80% +123%	50- 60 — — —	<200 ≥300 ≥400 ≥500 ≥600 nT	45 μT	>1 years	290 407	Unadjusted ORs, adjusted odds ratios (aORs), and 95% CI were calculated using unconditional logistic regression analysis	ELF-MF in bedrooms	Not applicable	Not applicable	0.42	[32]
54	Honey bees *Apis mellifera*, from 4 hives	Absolute wing flapping frequency	N/A N/A N/A	50 — —	0.1 1 7 mT	0 μT	15 min — — —	120	One-way and two-way ANOVA, Bonferroni post hoc test	Helmholtz coils (1 axis)	Ø 25 cm	Magnetometer, 3D map ELF-MF ≫ GMF	0.97	[131]
		Proportion of bees successfully trained to forage	−80%	—	0.1 mT	—	—	—						
55	Locust *Schistocerca gregaria*, 4–9 days, male and females	Absolute wing flapping frequency (slow flying insects)	+20% +5% +10%	50 — —	0.1 1 7 mT	<10 μT — —	10 min — —	162 — —	Kruskal–Wallis test as the data failed the Brown–Forsythe test, one-way and two-way ANOVA	Helmholtz coils (1 axis)	Ø 25 cm	Magnetometer, 3D map ELF-MF ≫ GMF	0.42	[132]
		Absolute wing flapping frequency (fast flying insects)	−5% −15% −20%	50 — —	0.1 1 7 mT	— — —	— — —	— — —						
56	Rats Sprague–Dawley, 200–250 g, age 8 weeks	Body mass	N/A N/A N/A	50 — —	30 100 500 μT	<10 nT — —	24 weeks—	30 — —	One-way ANOVA	Helmholtz coils	2000× 700× 2000 mm	Magnetometer, 3D map	0.42	[222]
		Water consumption	N/A N/A N/A	— — —	— — —	— — —	— — —	— — —						
		Count of the red blood cells	N/A N/A N/A	— — —	— — —	— — —	— — —	— — —						
		Protein expression: alanine transaminase,	N/A N/A N/A	— — —	— — —	— — —	— — —	— — —						
		aspartate aminotransferase	N/A N/A N/A	— — —	— — —	— — —	— — —	— — —						
		Concentration of micro- and macroelements: Cr	N/A N/A N/A	— — —	— — —	— — —	— — —	— — —						
		Ca^2+^	N/A N/A N/A	— — —	— — —	— — —	— — —	— — —						
		Mg^2+^	N/A N/A N/A	— — —	— — —	— — —	— — —	— — —						
		Blood urea nitrogen	N/A N/A N/A	— — —	— — —	— — —	— — —	— — —						
		Ultrastructure of the kidneys	N/A N/A N/A	— — —	— — —	— — —	— — —	— — —						
		Ultrastructure of the liver	N/A N/A N/A	— — —	— — —	— — —	— — —	— — —						
		H_2_O_2_ concentration	N/A N/A N/A	— — —	— — —	— — —	— — —	— — —						
		NO concentration	N/A N/A N/A	— — —	— — —	— — —	— — —	— — —						
		Catalase activity	N/A N/A N/A	— — —	— — —	— — —	— — —	— — —						
		SOD activity	N/A N/A N/A	— — —	— — —	— — —	— — —	— — —						
57	Sunflower and wheat seedlings	Fresh biomass of sunflowers: Whole plant, Shoots, Roots	+12% +15% +5%	16.6 — —	20 μT — —	~45 μT — —	12 days — —	6 — —	Kruskal–Wallis test	Helmholtz coils (1 axis)	Ø 60 cm	Magnetometer, oscilloscope 1 point, temperature variation <0.1%	0.42	[150]
		Fresh biomass of wheat seedlings (whole plant)	−50%	—	—	—	—	—						
58	Human Electric train drivers, 40–55 years old, men	Heat rate	−5%	16.6	1.5 μT	38 μT	24 h	7	Student’s *t*-test (pilot study)	Workplace	Not applicable	Not applicable	0.42	[223]
		HRV: LF HF	+6% +5%	— —	— —	— —	— —	— —						
59	Cardiomyocytes (hiPS line)	Electrical response to Verapamil	N/A	50	400 mT	0 mT	60 s	200	Student’s *t*-test	Helmholtz coils (1 axis) iron shield	50 × 50 cm	Magnetometer, 1 point, variation < 5%	0.98	[224]
60	Human cord blood cells CD34+ pluripotent stem cells	Myeloid differentiation Lymphoid differentiation	N/A N/A	50 —	300 mT	45 μT —	35 days —	4 —	Student’s *t*-test	Helmholtz coils (1 axis)	50 × 50 cm	Magnetometer, 1 point, variation < 5%	0.42	[173]
61	Mice BALB/c, 22–25 g Peritoneal neutrophils	Membrane peroxidation	+10.2%	1 +4.4 +16.5	600+ 100+ 160 nT	42 μT	1 h	3	Student’s *t*-test	Helmholtz coils (2 axes)	Ø 120 cm	Magnetometer, 1 point, variation < 2% GMF~42 μT TVMF 50 Hz 15–50 nT	0.18	[126]
		fMLF-induced ROS generation	+200%	—	—	—	—	—						
62	Mice CD-1, males, 22–25 g Peritoneal neutrophils	fMLF-induced ROS generation after cell treatment	+36%	12.6+ 48.5	100 nT	60 μT	1 h	3	Mann–Whitney test (continuity correction) Benjamini–Hochberg’s correction	Solenoid in a shell made of soft magnetic material	Ø 18 × 36 cm	Magnetometer, 1 point, variation TVMF 50 Hz <5 nT, SMF <10 nT GMF ~44 μT TVMF 50 Hz 15–50 nT	0.49	[127]
63	Mice BALB/c Age 8–10 weeks (25–27 г) Ehrlich ascitic carcinoma	TNF-α secretion: macrophages	−19%	(5.10+ 5.26+ 5.91+ 6.26+ 6.31+ 6.98)	100 nT	60 μT	28 h	30	Student’s *t*-test	Helmholtz coils (2 axes)	Ø 140 cm	Magnetometer, 1 point, variation <2% GMF ~37 μT	0.4	[128]
		fMLF-induced generation of ROS after addition of MF-treated water	+66%	—	—	—	—	—						
		TNF-α secretion by macrophages	+270%	—	—	—	—	—						
		TNF-α secretion by T-cells	+180%	—	—	—	—	—						
		TNF-α secretion by whole blood	+400%	—	—	—	—	—						
		IFN-γ secretion by macrophages	+200%	—	—	—	—	—						
		IFN-γ secretion by T-cells	+190%	—	—	—	—	—						
		IFN-γ secretion by whole blood	+90%	—	—	—	—	—						
		Tumor size	−40%	—	—	—	—	—						
		Survival rate at 50 days	+900%	—	—	—	—	—						
64	Mice Strains Tg and OBE (model of familial and sporadic Alzheimer’s disease) of the C3H and SO lines (appropriate controls)	Spatial memory test (Morris water maze): Tg, C3H, OBE, SO	+25% +25% N/A +25%	0.38+ 4.88 — —	80 nT — — —	42 ± 0.1 μT — —	40 h — — —	5 — — —	One-way ANOVA, *t*-test	Helmholtz coils (1 axis)	Ø 140 × 70 cm	Magnetometer, oscilloscope 1 point, variation < 1% TVMF 50 Hz 20–40 nT GMF~37 μT	0.4 [144]	
		Brain Aβ amyloid concentration: Tg, OBE	−25% −50%	— —	— —	— —	— —	— —						
65	Spinach *Spinacia oleracea* 4–5 weeks, insulated membranes	Ca^2+^ permeability	−6% +4% −9% −4% +9% +15% +4% −5% +5% +5% +1% −4% −1%	9 16.7 20 25.5 — — — — 30 40 50 60 80	25.9 μT — — 20.3 21.0 21.7 22.4 25.9 μT — — —	37 μT — — 29 30 31 32 37 μT	1 h — — — — — — — — — — — —	5	Student’s *t*-test	Helmholtz coils (2 axes)	-	Magnetometer, oscilloscope 1 point, variation <2.5%	0.42	[202]
66	Granulocytes differentiated from polypotent CD34+ umbilical cord blood cells	Cell death	+50%	50	1 mT	~1 nT	72 h	3	Wilcoxon rank-sum test	Helmholtz coils (1 axis) in μ-metallic chamber	15 × 15 cm	Magnetometer, oscilloscope 1 point, variation < 1%, GMF shielded with μ-metal chamber	0.97	[125]
		Apoptosis	+20%	—	—	—	—	—						
		Length of cell cycle phases	N/A	—	—	—	—	—						
		Proportion of genes with increased expression	+2%	—	—	—	—	—						
		Proportion of genes with reduced expression	+1.5%	—	—	—	—	—						
		DNA methylation	−5%	—	—	—	—	—						
67	Umbilical Cord Blood Lymphocytes	Cell viability	−15% −16%	7.8 —	6.6 12 μT	4 μT	72 h	3	ANOVA, post hoc Fisher LSD	Coils (2 axes)	20 × 20 cm	Magnetometer, 3D map, variation < 8%, GMF 33.6–38 μT, GMF shielded with μ-metal chamber	1.15	[225]
68	Cell line U251	Proliferation rate	+80%	7–21	24 μT	126 μT	72 h	3	ANOVA	Coils (2 axes)	20 × 20 cm	Magnetometer, 3D map, variation < 1 μT GMF 33–38 μT	1.14	[226]
69	*E. coli* strains AB1157 and EMG2	Anomalous viscosity time dependencies (AVTD) is strains: AB1157	+26% +23% +21%	16 30 64	21 μT — —	43 μT — —	15 min — —	3 — —	Student’s *t*-test	Helmholtz coils	Ø 17.6 cm	Magnetometer, one point, variation SMF < 2%, TVMF < 5%	book 0.72	[227,228]
		EMG2	+26% +21% +18%	16 28 55	— — —	— — —	— — —	— — —						
70	Wheat *Triticum aestivum* Control and Drought Conditions	Fresh, Control, Drought	N/A +90%	14.3 —	18 μT —	52 μT —	12 days —	3 —	Student’s *t*-test	Helmholtz coils (1 axis)	Ø 20 cm	Magnetometer, one point	0.79	[151]
		Length: Control, Drought	N/A +15%	— —	— —	— —	— —	— —						
		Leaf Area: Control, Drought	N/A +80%	— —	— —	— —	— —	— —						
		Photosynthesis efficiency: Control, Drought	N/A +60%	— —	— —	— —	— —	— —						
		Water content: Control, Drought	N/A +95%	— —	— —	— —	— —	— —						
71	*Bacillus Iicheniformis* α-amylase immobilized on superparamagnetic particle	Enzyme activity	+28% +27%	5 7	12 mT —	50 μT —	30 min	3	Student’s *t*-test	System of 4 coils	10 × 10 cm	Magnetometer, one point	0.79	[218]
72	Fruit fly *Drosophila melanogaster* wild type, eggs	Mortality: eggs, larvaem, pupae, adult	+350% N/A +140% −33%	50 — — —	1 mT — — —	40 μT ^3^ — — —	48 h — — —	1000 — — —	Two-way ANOVA	Helmholtz coils (1 axis)	Ø 17 cm	Magnetometer, oscilloscope one point ELF-MF— GMF	0.42	[156]
73	Fruit fly *Drosophila melanogaster* wild type and Cy/Pm mutants (curly wings and plum-colored eyes) hybrids	Percent of frequency of recessive lethal illnesses	N/A N/A	50 —	0.5 5 mT	45 μT —	500 days (40 generations)	>100 —	ANOVA, Chi-square test of goodness-of-fit, Bartlett’s test	Helmholtz coils (2 axes)	Ø 40 cm	Magnetometer, one point Induction ELF-MF— GMF	0.43	[157]
		Average viability	−15% −20%	— —	0.5 5 mT	— —	— —	— —						
74	Fruit fly *Drosophila melanogaster* wild type, eggs	Embryo survival	+25% +30%	50 50	5 μT 40 μT	200 nT 200 nT	3 h	30 30	ANOVA, Student–Newman–Keuls, and Dunnett’s post hoc test	Helmholtz coils (1 axis)	-	Magnetometer, one point	1.25	[161]
75	Fruit fly *Drosophila melanogaster* wild type, adult	Eggs from Petri dishes: F1, F2, F3	+100% −30% −60%	50 — —	2 mT ↕ —	48 — —	3 days	5	Student’s *t*-test	Helmholtz coils (1 axis)	Ø 17 cm	Magnetometer, one point TVMF variation < 0.2 mT GMF (not described) Temperature variation < 1.5 °C	0.43	[158]
		Mature individuals: F1, F2, F3	+22% −30% −60%	— — —	— — —	— — —	— — —							
		Number/% of dead eggs: F1, F2, F3	+480% +260% +160%	— — —	— — —	— — —	— — —							
76	Fruit fly *Drosophila melanogaster* wild type, adult	Number of F1 pupae per maternal insect Ovarian DNA fragmentation (TUNELpositive eggs):	−2.9% −3.7% −4.3%	50 — —	0.1↕ 1.1↕ 1.2 mT↕	GMF — —	48 h — —	12	ANOVA, Pearson’s correlation analysis	Helmholtz coils (1 axis)	Ø 25 cm	Magnetometer, oscilloscope, spatial distribution, *E* components 0.13 1.43 2.72 V/m Temperature variation < 1 °C	0.65	[159]
			+5.7% +6.7% +7.5%	— — —	0.1↕ 1.1↕ 1.2 mT↕	— — —	— — —	— — —						
77	Zebrafish *Danio rerio* embryos	Mortality	N/A N/A N/A	50 — —	0.2 0.4 0.8 μT	13 μT — —	96 h — —	100 — —	ANOVA, LSD test	Helmholtz coils (1 axis)	100× 100× 50 cm	Magnetometer, spatial distribution, variation SMF < 20 nT, TVMF < 1%	0.73	[160]
		Ebryo malformation	N/A N/A N/A	— — —	0.2 0.4 0.8	— — —	— — —	— — —						
		Heart rate 36 h of development	−5% −15% −12%	— — —	0.2 0.4 0.8	— — —	— — —	— — —						
		Hatching rate, 48 h of development	−60% −60% −50%	— — —	0.2 0.4 0.8	— — —	— — —	— — —						
		54 h of development	−60% −80% −90%	— — —	0.2 0.4 0.8	— — —	— — —	— — —						
		60 h of development	−8% −10%	— —	0.4 0.8 mT	— —	— —	— —						
		Gene expression: *caspase-3*	+20% +20% +20%	— — —	0.2 0.4 0.8 mT	— — —	— — —	— — —						
		*caspase-9*	+35%	—	0.8 mT	—	—	—						
78	Glioblastoma cell line U251 and breast cancer MDA-MB-231 cell line	U251 cell proliferation rate	+12% +14% −60% −55% −40% −30% −40%	7+14+20 7.8 — — — —	6 24 6 10 13 17 24	>17 μT — — — — — —	7 days — — — — — —	3 — — — — — —	ANOVA, Dunnet’s post hoc test	Perpendicular coils	~130× 90 mm	Magnetometer, oscilloscope, 3D map, variation SMF < 2 μT, TVMF <100 nT GMF < 2% GMF 41.7 μT	1.14	[226]
		MDA-MB-231 cell proliferation rate	−10% −15% −20%	— — —	6 10 13 μT	— — —	— — —	— — —						
79	Human SH-SY5Y neuroblastoma cells and mouse primary cortical neurons (PCNs)	PCNs cells: p53 fold change	−10% −20%	50 —	1 mT —	300 nT —	48 h —	3 —	Two-way ANOVA, Friedman test	Helmholtz coils (1 axis)	38 × 12 cm	Magnetometer, 3D map, TVMF and SMF variation < 5%, temperature variation < 0.2%	1.33	[208]
		SH-SY5Y cells: p53 fold change	+30%	—	—	—	48 h	—						
		Proportion of 5-metylcitosine in DNA	+50%	—	—	—	4 h	—						
		Superoxide regeration	+80%	—	—	—	24 h	—						
		H_2_O_2_ regeration	+120%	—	—	—	24 h	—						
		Expression of *Btg4 * (cell cycle regulator): control, DAG-treated cells	70% N/A	— —	— —	— —	6 h —	— —						
		Mitochondrial potential	−30% −20%	— —	— —	— —	24 h 48 h	— —						
		Alpha-synuclein expression	+25%	—	—	—	48 h	—						
		Alpha-synuclein aggregation	+30%	—	—	—	—	—						
		Levels of differentiation regulators miR-34b	−25% −80% −90%	— — —	— — —	— — —	24 h 48 h 72 h	— — —						
		miR-34c	−30% −25%	— —	— —	— —	48 h 72 h	— —						
80	Human SH-SY5Y neuroblastoma cells	DHE-detected ROS generation (superoxide)	+20% +25% +40%	50 — —	1 mT — —	300 nT — —	24 48 72 h	3 — —	Two-way ANOVA, Friedman test	Helmholtz coils (1 axis)	38 × 12 cm	Magnetometer, 3D map, TVMF and SMF variation < 5%, temperature variation < 0.2%	1.33	[209]
		DCF-detected ROS generation (H_2_O_2_)	+30% +70% +40%	— — —	— — —	— — —	24 48 72 h	— — —						
		Thiols content (antioxidants)	−20% −25% −15%	— — —	— — —	— — —	24 48 72 h	— — —						
		MPP+ toxin induced: proliferation inhibition	+20%	—	—	—	72 h	—						
		Cell death	+100%	—	—	—	—	—						
		Apoptosis	+400%	—	—	—	—	—						
		Caspase 3/7 activation	+200%	—	—	—	—	—						
81	Calves, adult	Melatonin concentration in saliva: winter, summer	−50% +25%	50 —	400 nT	49 μT —	80 days —	80 —	Multivariate general linear mixed model	Custom-built coil, TVMF variation < 10 nT	-	Magnetometer, one point	0.97	[198]
82	Immortalized nontumorigenic human keratinocytes HaCaT	Cell number,	−30%	60	1.5 mT	0.47 μT	144 h	3	Student’s *t*-test	Helmholtz coil	Ø 37 cm	Magnetometer, spatial distribution, variation, TVMF < 4.4%, SMF < 30 nT, Temperature variation < 0.3 °C, pH of culture medium variation < 0.02	0.89	[204]
		Number of colonies	−20%	—	—	—	—	—						
		Cell cycle phase duration: G0/G1, S, G2/M	+30% −60% −10%	— — —	— — —	— — —	— — —	— — —						
		Proteins levels: phospho-Chk2 (Thr68),	+100%	—	—	—	—	—						
		p21	+100%	—	—	—	—	—						
83	Immortalized COS7, CHO, HB2, and MEF, transformed MDA-MB-231 (MDA), HeLa, and PC3, Jurkat and REH cell lines	pERK amount in cells CHO	+50% +200%	50 —	7 μT 1 mT	10 nT —	71 min —	3 —	Student’s *t*-test	sXcELF ELF-MF exposure system	No discribed	Magnetometer, one point	0.83	[205]
		MEF	+500% +450%	— —	7 μT 1 mT	— —	— —	— —						
		HB2	+400% +450%	— —	7 μT 1 mT	— —	— —	— —						
		COS7	+200% +450%	— —	7 μT 1 mT	— —	— —	— —						
		HeLa	+80% +80% +90% +200% +350%	— — — — —	7 μT 15 μT 50 μT 1 mT 10 mT	— — — — —	71 min 15 min — — —	— — — — —						
		Juncat	+100% +200%	— —	7 μT 1 mT	— —	— —	— —						
		p-p38 MAPK amount in cells COS7	N/A N/A	— —	7 μT 1 mT	— —	70 min —	— —						
		HeLa	N/A N/A	— —	7 μT 1 mT	— —	— —	— —						
		pJNK amount in cells COS7	N/A N/A	— —	7 μT 1 mT	— —	— —	— —						
		HeLa	N/A N/A	— —	7 μT 1 mT	— —	— —	— —						
		pAKT amount in cells COS7	N/A N/A	— —	7 μT 1 mT	— —	— —	— —						
		HeLa	N/A N/A	— —	7 μT 1 mT	— —	— —	— —						
84	Wistar rats aged 8 weeks old, healthy or with modeled Alzheimer’s disease, hippocampal neurons	Phosphorylation level of NF-κB	+120% +40% +40% N/A	50 — — —	400 μT — — —	35 μT	6 h 7 14 28 days	3	ANOVA, Levene’s test for homogeneity of variances	Helmholtz coils (1 axis)	140 × 70 cm	Magnetometer, one point, variation, TVMF <20 μT Background TVMF 50 Hz <100 nT, GMF not described	0.79	[207]
		Phosphorylation level of IKK	+40%	—	—	—	6 h	—						
		Expression level of RKIP and TAK1	−25% −20% −20%	— — —	— — —	— — —	14 days 6 h 14 days	— — —						
		RKIP/TAK1 interaction	−80% −80% −75% N/A	— — — —	— — — —	— — — —	6 7 14 h 28 days	— — — —						
		Behavior Morris water maze test	+30% +25% +25% +25%	— — — —	— — — —	— — — —	6 7 14 h 28 days	— — — —						
		Alzheimer’s disease effect in model rats	−80% −60% −75% −90%	— — — —	— — — —	— — — —	6 7 14 h 28 days	— — — —						
85	Flax *Linum bienne* upper segments of stems without leaves, 2.5 cm long	Deviation of the apical end of a segment from the horizontal plane (gravitropism)	+15% +20% +32% +40% +44% +36% +29% +4%	35.8 — — — — — — —	32.6 41.9 60.5 74.4 83.7 97.7 130.2 158.1 μT	46.5 — — — — — — —	2 h	20	Student’s *t*-test	Helmholtz coils	Ø 30 cm	Magnetometer, one point, TVMF 50 Hz 5 nT	0.18	[55]
86	Chromaffin cell cultures from rats	Proportion of cells with neurite-like growth	+220%	60	0.7 mT	50 μT	28 h	6	Student’s *t*-test	Helmholtz coil (1 axis)	Ø 18.32 cm	Magnetometer, spatial distribution	0.99	[181]
		Neurite length	+110%	—	—	—	—	—						
		Change in potential induced by Ca^2+^ curren	+110%	—	—	—	—	—						
		KCl-evoked catecholamine release	+700%	—	—	—	—	—						
87	tT20 D16V neuronal cells	Ca^2+^ influx	+30%	50	2 mT	44 μT	48 h	500	Student’s *t*-test	Solenoid	Ø 10 cm	Magnetometer, one point E = 12 V/m, temperature variation < 0.3 °C, GMG (not described)	0.42	[182]
		Intracellular pH	−0.2 pH units	—	—	—	—	—						
		Neurofilament-positive cells count: control, Nifedipine treated (Ca^2+^ channels antagonist),	+260% −15%	— —	— —	— —	— —	3 —						
		Synaptophysin protein-positive cell count	+3000%	—	—	—	—	—						
		*NF-200* gene expression	+100%	—	—	—	—	—						
88	Neural stem/progenitor cells from the brain cortices of newborn mice	Beta-III-tubulin^+^ cells: 6 days, 12 days	+90% +90%	50 —	1 mT —	44 μT —	24 h —	90 —	Student’s paired and unpaired t-test	Solenoid	Ø 20 cm	Magnetometer and oscilloscope, one point, temperature 37.4 ± 0.1 °C (both control and sham incubators)	1.29	[175]
		MAP2^+^ cells count: 6 days, 12 days	+15% +20%	— —	— —	— —	— —	— —						
		Surface expression of Ca(v)1.2 channel	+100%	—	—	—	—	—						
		Surface expression of Ca(v)1.3 channel	+100%	—	—	—	—	—						
		Spontaneous Ca^2+^ transients frequency	+100%	—	—	—	—	—						
		Spontaneous Ca^2+^ transients amplitude	+20%	—	—	—	—	—						
		KCl-induced Ca^2+^ transients frequency	+25%	—	—	—	—	—						
		Amplitude of KCl-induced Ca^2+^ transients	+30%	—	—	—	—	—						
		pCREB+ cells count	+400%	—	—	—	—	—						
89	CHO-K1 cells transfected Kv1.3 channel	Whole-cell Kv1.3 steady-state conductance	+5% +10%	20 —	268 902 μT	44 μT —	1 min —	92 44	Wilcoxon signed-rank test	Solenoids	Ø 88 mm	Magnetometer, one point	0.4	[176]
90	CA1 pyramidal neurons of young Sprague–Dawley rats	Maximum current density of I_Na_ (modulus of pA/pF)	+29% +32% +38% +72% +80% +94% +147% +136% +103% +10% +71% +86% +380% +345% +312% +407% +413% +441%	15 — — — — — — — — 50 — — — — — — — —	0.5 — — 1 — — 2 — — 0.5 — — 1 — — 2 — —	50 μT — — — — — — — — — — — — — — — — —	10 20 30 10 20 30 10 20 30 10 20 30 10 20 30 10 20 30	5 — — — — — — — — — — — — — — — — —	ANOVA on ranks, Tukey’s post hoc test	Coils system (1 axis)	18 × 69 mm	Magnetometer, spatial distribution, TVMF variation < 8%, ELF-MF— GMF	0.4	[177]
		Maximum current density of I_k_ (modulus of pA/pF)	−30% −40% −30% −25% −40% −30% −30% −40% −25% −35% −20% −50% −75% −20% −40% −55%	15 — — — — — — — — — — — — — — —	0.5 — 1 — — 2 — — 0.5 — 1 — — 2 mT — —	— — — — — — — — — — — — — — — —	20 30 10 20 30 10 20 30 20 30 10 20 30 10 20 30	— — — — — — — — — — — — — — — —						
91	Neurogenic tumor cell lines (U251, A172, SH-SY5Y) and primary cultured neurogenic cells from rat embryos (astrocytes, microglia, cortical neurons)	γH2AX foci formation (all cells)	N/A	50	2 mT	50 μT	24 h	3	Student’s *t*-test	Exposure system (sXc-ELF) on base of Helmholtz coils	-	Magnetometer, oscilloscope, one point, temperature variation <0.1°C	0.57	[229]
		cell cycle phases proportion (all cells)	N/A	—	—	—	—	—						
		cell viability (all cells)	N/A	—	—	—	—	—						
		total dendritelength	N/A	—	—	—	—	—						
		average dendrite branch length	N/A	—	—	—	—	—						
		average number of branches	N/A	—	—	—	—	—						
92	Children, boys and girls, healthy or with leukemia	Risk of cancer development: leukemia	+70%	60	0.1–10 μT	50 μT	10 years	936	Chi-squared test	Epidemiological study	Not applicable	Not applicable	1.81	[230]
		lymphoma	+100%	—	—	—	—	—						
		nervous system tumors	+80%	—	—	—	—	—						
		other tumors	+90%	—	—	—	—	—						
93	Humans, adult, men and women, healthy or with leukemia	risk of cancer development	+64% +43%	60 —	0.25 0.12 μT	50 μT —	7 years —	56 134	Chi-square test	Epidemiological study	Not applicable	Not applicable	1.81	[231]
94	Children, boys and girls, <16 years old, healthy or with leukemia	Risk of cancer development: all cancer	+50% +20% +30%	50 — —	0.1–0.2 0.2–0.3 >0.3	53 μT — —	<15 years — —	127.383 — —	Spearman rank correlations, confidence intervals, logistic regression model Mantel extension technique	Living <300 m from any of the 220 and 400 kV power lines	Not applicable	Not applicable	1.81	[232]
		leukemia	+110% +50%	— —	0.1–0.2 0.2–0.3	— —	— —	— —						
		lymphoma	+280% +30%	— —	>0.3 0.2–0.3 μT	— —	— —	— —						
95	Humans, adult, men and women, healthy or with cancer	Risk of cancer development: acute myeloid leukemia	+70%	50	>0.2 μT	53 μT	10–15 years	>300	Spearman rank correlations, confidence intervals, logistic regression model Mantel extension technique	Living <300 m from any of the 220 and 400 kV power lines	Not applicable	Not applicable	1.96	[233]
		chronic myeloid leukemia	+70%	—	—	—	—	—						
		central nervous system tumors	N/A	—	—	—	—	—						
96	Humans, adult, men, electric utility workers, healthy or with cancer	Risk of cancer development: all hematopoietic malignancies,	+23% +23%	60 —	>3.2 ^3^ >7	55 μT —	years ^2^ —	31.543 —	X^2^ test	Ontario electric utility power lines	Electric fields were >172 V/m or >345 V/m, respectively	Not applicable	1.81	[234]
		non-Hodgkin’s lymphoma	+27% +29%	— —	>3.2 >7	— —	— —	— —						
		acute nonlymphoid leukemia	+93% +187%	— —	>3.2 >7	— —	— —	— —						
		acute myeloid leukemia	+287%	—	>7	—	—	—						
		chronic lymphoid leukemia	N/A N/A	— —	>3.2 >7	— —	— —	— —						
		malignant brain tumors	N/A N/A	— —	>3.2 >7	— —	— —	— —						
		benign brain tumors	+483% +464%	— —	>3.2 >7	— —	— —	— —						
		malignant melanoma	N/A N/A	— —	>3.2 >7	— —	— —	— —						
		stomach cancer	+123%	—	>3.2	—	—	—						
		lung cancer	+100% +22%	— —	>7 >7 μT	— —	— —	— —						

^1^—Unless otherwise indicated in the publication, magnitude of GMF induction was indicated according to the World Magnetic Model map (https://www.ncei.noaa.gov/products/world-magnetic-model access on 10 October 2023), ^2^—unless otherwise stated, the exposure was counted for 7 years, as was shown in the work [231], ^3^—cumulative level μT/years, ——repeated values, N/A—no effect detected, ↕—vertical margin (if specified), ↔—horizontal margin (if specified), ↔↕—combination of vertical and horizontal margins (if specified), ++—moderate increase in parameter (qualitatively), +—slight increase in parameter (qualitatively). If the incubation consisted of several sessions, then the total exposure time during the experiment is indicated. SJR—scientific journal rankings (https://www.scimagojr.com/journalrank.php, access on 16 October 2023).

### 3.3. Effects of Anthropogenic Fields (Epidemiological Studies)

**Figure 5:**
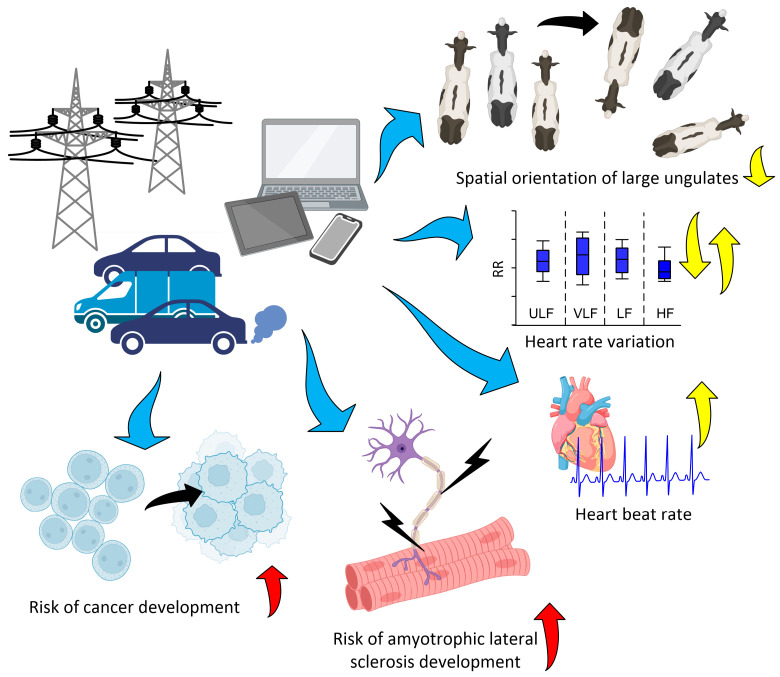
The main effects of background anthropogenic ELF-MFs on humans and animals. The directions of the arrows indicate the direction of the effect: up—increasing the parameter, down—decreasing the parameter. The color indicates the expected impact of the effect on the organism being studied: yellow—difficult to assess, red—negative. References are presented in the text and Table 1.

The effects of background EMFs largely depend on the animal species (Figure 5, Table 2). ELF-MFs with characteristics close to the background EMF of cities *f*(50)b(30–500)B(0.001)t(24 weeks), in the case of rats, even with long-term exposure did not affect body weight, water consumption, leukocyte blood count, expression of aminotransferases, Ca^2+^ concentrations and Mg^2+^ in the blood, or functions and structure of the kidneys and liver [222]. Birds, large ungulates, and humans are more sensitive to EMFs (see below).

Background MFs generated near high-voltage power lines *f*(50–60)b(5–15) disrupt the natural spatial orientation of large ungulates: cows and roe deer [64].

In some works, it has been suggested that a background ELF-MF *f*(60)b(>0.3)B(GMF)t(years) generated in cities may be a potential risk factor for developing leukemia and B-line acute lymphoblastic leukemia in children by one and a half to three times compared with children from “magnetic-favored” regions. An association was found between exposure >0.4 µT and childhood leukemia compared to ELF-MF exposure at doses below 0.1 µT [32,69,70]. The proximity of children’s families to power lines and parental occupational exposure to ELF-MFs at specific times before or during pregnancy were inconsistent but may be associated with childhood leukemia [235]. Towards the end of the previous century, it was found that residing in residences equipped with wiring of a high current configuration *f*(60)b(0.1–10)B(45)t(7–10 years) led to a higher likelihood of cancer (leukemia, lymphoma, etc.) in children, with risks increasing by 70–100% and 40–60% for children and adults, respectively [230,231]. Electric utility power line workers with cumulative exposures >3.2 μT-years and >345 V/m-years were found to have increased relative risks of developing hematopoietic malignancies, brain tumors, and lung cancer [234]. Living within 300 m of high-voltage power lines (220 and 400 kV) is associated with increased risks of leukemia and lymphoma [232,233,236]. It is important to recognize that many factors contribute to the risk of developing cancer. The magnetic environment is not a major risk factor. The relationship between the likelihood of developing cancer and exposure to MFs is currently being very actively researched and refined [237].

Background ELF-MFs with an average daily induction of >300 nT doubles the risk of developing leukemia in children, while a considerable proportion of children in large cities are exposed to just such EMFs [238]. Unfortunately, it is difficult to ensure correct randomization in this type of epidemiology study [32]. The so-called “wire code” paradox is considered an additional risk factor for the development of childhood leukemia. It states that for weak TVMFs with an induction of 0–0.1 μT, the effect on the risk of developing leukemia is comparable to that of stronger ones >0.3 μT [69]. However, in other studies, the presence of this phenomenon was not confirmed [70].

Long-term exposure to an elevated ELF-MF *f*(50–60)b(>0.3)(GMF)t(years) among railway workers (drivers) appears to be a risk factor for developing amyotrophic lateral sclerosis [65].

Among the “fast” effects of the background ELF-MF of the working zone *f*(16.6)b(1.5)t(1 day), a decrease in heart rate and an increase in heart rate variability were found, both in the low-frequency and high-frequency rhythms [223,239]. It is worth noting that the long-term consequences of the action of anthropogenic MFs on animals and plants are now beginning to be actively studied [240].

## 4. Potential Mechanisms of Action of Magnetic Fields

The search for the mechanisms of biological effects of MFs began at the end of the last century. During this time, slightly less than a dozen theoretical mechanisms of the action of MFs on living systems were proposed [23,53]. The targets of MFs can be molecules as a whole, protons, electron spins, spin-correlated pairs of radicals, quantum rotations of molecular groups inside proteins, and orbital magnetic moments [76,241,242,243,244]. The quantum mechanisms of these phenomena are described in detail in [23,53]. Some of them were partially confirmed in experiments (see below) [245,246,247,248].

The most obvious mechanism is the thermal effect of MFs [249,250,251,252]. This mechanism explains the effects on biological systems due to changes in the rates of chemical reactions according to the principles of thermodynamics [244]. However, the thermal effects of MFs at a frequency of <100 MHz require very high induction values of ~10 mT or more [253,254], which significantly exceeds the induction values that can have biological effects (Table 1 and Table 2).

Despite this, EMFs with frequencies of 50 and 60 Hz induce cellular stress responses comparable to the response to heating [183]. The energy absorbed by the system when heated to +5.5 °C is 2.3 × 10^7^ J/m^3^. The energy absorbed by the system upon exposure to MF of 8 μT is 2.6 × 10^−7^ J/m^3^, which is 14 orders of magnitude lower, but the transcriptional response of the cell to both of these influences is comparable in order of magnitude [193].

Therefore, the search for possible “non-thermal” mechanisms is central to the study of the biological effects of MFs at environmental intensities.

Another mechanism often implemented in inanimate systems is eddy currents induced by MFs and the deflection of charged particles by the Lorentz force [255]. Data with a high induction MF > 1 T and different directions showed that the direction of the MF can affect the rate of synthesis of chiral molecules in the example of DNA, as well as the rate of proliferation of cell lines [256]. This mechanism is theoretically applicable for variable MFs but requires significant induction values > 20 mT at a frequency of 50 Hz [194]. Thus, to generate eddy currents in a living cell sufficient for biological effects, it is necessary to apply an MF with an induction 500–1000 times higher than the GMF [52]. In the case of work with TVMF inductions slightly exceeding the magnitude of the GMF, the effect of the Lorentz force is orders of magnitude lower than electric diamagnetism, therefore it cannot be considered the main effector of biological effects in magnetobiological studies [23].

Experimental data indicate that in several cases the impact of an MF is amplified within certain frequency and amplitude “windows” depending on several physical parameters [30,42,48,57,149,167,168,171,202,257,258]. Such frequency and amplitude “windows” can be explained using ion cyclotron resonance (ICR). The original idea of using ICR to explain magnetobiological effects was proposed by Liboff [259,260,261]. The hypothesis assumed that calcium and potassium ions are used to enhance transport through membrane ion channels. The hypothesis was based on a large number of experimental facts in which biological evidence showed that the effects had resonance-like dependences on frequencies close to cyclotron frequencies (~10–70 Hz) of biologically relevant ions in magnetic fields close to the geomagnetic field (10–150 μT). For example, the recorded effects of ELF-MFs were of a resonance-like nature, which often coincided with the cyclotron frequencies of ions, for example, Ca^2+^ [149,171,259,262]. Many enzymes, including endonucleases, topoisomerases, and polymerases, contain biologically significant ions Mg^2+^, Ca^2+^, Zn^2+^, etc., which are important for the stability of the conformation of these proteins and their enzymatic activity. The ions are often bound in special protein pockets by amino acids such as histidine or cysteine [263,264]. This type of binding is dynamic and is characterized by a specific retention time of ions within proteins. The absence of ions in protein pockets leads to significant changes in protein conformations and enzyme activity.

Many attempts have been made to explain the mechanisms involving ions as MF receptors [259,261,265,266,267,268,269,270]. According to models, ELF-MFs affect cells through exposure to non-hydrated ions inside protein cavities, if the exposure parameters (frequency and magnetic induction of AMF and induction of SMF) are tuned to these ions [53,149,271]. Based on these models, “effective” impact parameters can be obtained analytically. Effective or “resonant” frequencies and effects, depending on the induction of constant and alternating MFs, are calculated from the equations:

$$f_{n}=\left(\frac{1}{2\pi n}\right)\left(q/m\right)B_{DC},\;p=J_{n}\left(knB_{AC}/B_{DC}\right)\;\left(n=1,2,3\ldots \right)$$

where *p* is magnetobiological effect level, *q* and *m* are the charge and mass of the ion, respectively, and *B_AC_* and *B_DC_* are the induction of AMF and SMF. AMF is collinearly aligned with the SMF. *n* is the resonance index number. *J_n_* is the Bessel function of the *n*-th order [271,272]. The coefficient *k* in the argument of the Bessel function is equal to one for Lednev’s model [272] and two for Blanchard and Blackman [271]. The first maximum of the effect is observed at

$$B_{AC}/B_{DC}\approx \;1.8\;\mathrm{or}\;B_{AC}/B_{DC}\approx \;0.9,$$

respectively.

The above-mentioned models have been criticized from a physical point of view [53,273]. However, in biological experiments, quite convincing evidence of its applicability has been obtained. Using the example of the gravitropism of flax (*Linum bienne*) stalks, it has been shown that the maximum magnetobiological effect in some fields is achieved by an FLF-MF with the following characteristics *f*(35.8)b(32.6–158.1)B(46.5)t(2 h) at $B_{AC}/B_{DC}\approx$ 1.8 [55]. In another paper, it was shown that the greatest biological effect of an ELF-MF is achieved when the ratio of b/*f* = 0.9 [148]. The obtained quantitative data indicate the realization of resonance phenomena (Lednev’s model) in the action of TVMFs on living systems in vitro.

As mentioned above, polarized/coherent EMFs (including ELF-MFs) can change the modes of Ca^2+^, Na^+^, and K^+^ VGIC functioning [175,176,177]. One of the main mechanisms of this effect is the forced vibration of ions, due to which external MFs can change the interaction of ions with the channel’s sensor [245,246]. According to calculations, forced vibration in an external TVMF for a single ion and a channel can be realized at induction >2 μT and intensity >1 V/m in the frequency range 1–20,000 Hz for both uni- and divalent ions [245]. It is noteworthy that if the electric field is removed from the calculations, then for an “isolated” MF, biological effects can be realized only at inductions >50 μT for divalent ions and >15 mT for univalent ions. Experimental data on biological effects on ELF-MF ion channels with a frequency of 15–50 Hz and an induction of 0.5–2 mT [177] indicate the importance of the electric component of EMFs in inducing biological effects. It has been described in the literature that ELF-MF-induced loss of adequate VGIC functioning, in turn, can lead to increased ROS generation and subsequent DNA damage and other intracellular events [274,275,276]. The participation of VGICs may explain the presence of amplitude “windows” in which the biological effects of ELF-MFs are realized [277].

Further, both the classical approaches, e.g., related to irregular gating of ion channels by polarized or coherent EMFs [277], and the quantum mechanical approaches have been used to explain the frequency and amplitude windows. For example, the interference of angular ion-molecular states approach was developed by Binhi [53]. Quantum mechanics was used to substantiate the existence of the coherent clusters predicted in the Preparata models of quantum electrodynamics in condensed matter and the Del Giudice quantum field thermodynamics of water [278,279]. In these models, water has a two-phase state and is the main interface of interaction with the MF. The existence of a two-phase structure of liquid water was confirmed in works [280,281].

Despite some criticism of the cyclotron model, there is a significant amount of experimental data that corresponds to the formal predictions arising from models associated with cyclotron resonances [54,202,282]. Considering that there are many biologically significant ions in the cell, assessing the effective inductions of SMF, AMF, and the AMF frequencies seems to be a difficult task. In addition, it is necessary to take into account not only the cyclotron frequencies but also their harmonics and subharmonics, which may be involved in the response to the ELF-MF to estimate the effective amplitudes of the ELF-MF. Finally, the direction of the field is also important and the perpendicular components of the AMF/SMF must be taken into account in the models [166,169]. As a result, a clear algorithm for assessing effective AMF/SMF combinations for the biological effect of weak ELF-MFs has not yet been developed.

The Schumann resonance should be noted among the resonant phenomena in the Earth’s magnetosphere. Schumann resonance is the phenomenon of the formation of standing electromagnetic waves of extremely low frequencies (7.8, 14.1, and 20.3 Hz) between the Earth’s surface and the ionosphere [283,284]. On the one hand, the induction of these EMFs is extremely small ~1 pT [117]. This induction is several orders lower than the electromagnetic noise of the city in this frequency range [24]. On the other hand, in some of the studies, the effects were discovered at frequencies close to the Schumann resonance [135,149], which is also possible due to exposure to cyclotron resonances. Therefore, the 7.8 and 20.9 Hz described in these works are similar to the second subharmonic of cyclotron resonance frequencies of Ca^2+^, K^+^, or Zn^2+^ and near cyclotron resonance frequencies of Zn^2+^ in some conditions [135,149,187]. The frequency 14.1 Hz may be a resonance frequency of Mg^2+^ in some conditions [226]. On the other hand, the fundamental frequencies of the Schumann resonance are represented by 7.8 Hz, 14.1 Hz, 20.3 Hz, 26.4 Hz, and 32.5 Hz [285] and fall within the frequency ranges of theta (4–7 Hz), alpha (7–12 Hz), sigma (12–14 Hz), beta (13–30 Hz), and gamma (30–80 Hz) rhythms of human brain electrical activity [63,286,287,288,289,290,291]. A high similarity of the human EEG profile to low Schumann resonance frequencies has been described. In addition, high coherence of low-frequency rhythms was found between the EEGs of people whose EEG frequency characteristics were closest to the Schumann resonance [292].

In addition, there is data on the effect of GMF disturbances at the Moshiri Schumann resonance frequency 8.0 ± 0.5 Hz on cardiovascular system functioning and psychological well-being. The decrease in blood pressure and improvement of psycho-emotional state in 30% of the analyzed population was observed on days with increased geomagnetic disturbances at the Moshiri Schumann resonance frequency [117].

Another possible target of ELF-MFs in cells is magnetic nanoparticles. Nanoparticles of magnetite and maghemite have been found in many organisms [293]. In MFs comparable in induction to the GMF, the energy of a 100 nm magnetosome is many times higher than the *kT* activation energy of chemical reactions [294,295]. Nanoparticles fixed in tissues and the cytoskeleton in ELF-MFs may presumably deform nearby biological structures, possibly leading to biological effects. In addition, magnetic nanoparticles themselves create fairly strong MFs near their surface, up to 100 mT at a distance of ~100 nm [23]. However, magnetic effects are observed in cells, plants, and animals lacking nanoparticles [77]. In addition, the mechanism of magnetic nanoparticles does not describe the observed frequency and amplitude windows. For this reason, we believe that this mechanism cannot be the main one to explain most magnetobiological effects.

The next mechanism is the formation of spin-correlated radical pairs [296,297]. The radical pair mechanism is the most developed at the present time. It is one of the most studied and has a significant amount of experimental evidence [243,298]. Radical pairs are described in the regulatory proteins of plants and animal cryptochromes, as well as in the cone cells of migratory birds and ommatidia (“eyes”) of insects [299,300,301,302]. Certain magnetic conditions (changes in the direction and induction of the MF) can cause singlet–triplet (S-T) conversion in radicals, which initiates conformational changes in cryptochromes and triggers further signaling events [243,303,304]. In a single radical pair, MF with an induction of 0.1–100 μT will produce a weak magnetic response that is unlikely to exceed 0.1% of the baseline [305]. However, numerous duplications and ordered arrangements are the mechanism for increasing the sensitivity of radical pairs in living organisms. Thus, the responses of all radical pairs are summed up and reach a sufficient amplitude to trigger signaling cascades (in the central nervous system in animals or transcriptional regulation in plants) [306,307].

This mechanism is well described in spin chemistry, where MFs with an induction of ~10 mT or more change the rates of some chemical reactions [244,303,308]. According to quantum calculations, MFs can influence the act of reaction via a change in the probability of rearrangement or the spatial distribution of the wave functions of electrons of interacting molecules [244,308]. An increase in H_2_O_2_ generation due to the formation of singlet oxygen during the S-T transition has been experimentally shown for SMFs with an induction of 1–7 T [309,310]. However, the energy of the S-T transition is orders of magnitude lower than the activation energy of a chemical reaction in ELF-MFs with inductions <50 μT field. Therefore, the ELF-MF data can only be considered as a regulator of the rate of a chemical reaction that has an activator [311]. Unfortunately, the mechanism of radical pairs has low-frequency sensitivity due to the short lifetime of the correlated state of spins (10^−9^ s, rarely 10^−7^ s) [305]. Therefore, frequency-dependent effects and effects of electromagnetic fields at environmental intensities are difficult to explain by radical pair mechanisms. The small lifetimes of radical pairs impose significant limitations on the magnitude of magnetic fields that can influence the singlet–triplet transition. Thus, a lifetime of ~200 ns increases the transition probability by 30% even in very low-intensity MFs comparable to the GMF, while for ~10 ns lifetimes, magnetic fields of much higher induction are required [312]. Another limitation is the size of the magnetobiological effect induced in the GMF without an amplification mechanism. As mentioned above, in vitro experimental confirmations work only with sufficiently strong magnetic fields from >10 mT. Even in this case, the maximum observed changes in the rate constant in biochemical reactions are only 10–60% [313]. Amplification mechanisms need to be employed for ELF EMFs at environmental intensities in order to be able to induce biological effects according to the radical-pair hypothesis. One possible amplification mechanism is via cryptochrome proteins found in the photoreceptors of birds [306,307]. Currently, the theory of the mechanism of spin interactions is being revised. In particular, the model involving radical triads rather than pairs has been developed for the implementation of magnetic biological effects [314]. In addition, approaches to improve the RPM model are discussed. For example, the inclusion in the calculations of resonance transitions between electronic and nuclear moments shows a high coupling to magnetic fields of 30–65 μT [315]. The RPM may be a special case of a more extensive mechanism, which will be discussed below.

According to Binhi [305] the interference of angular ionic-molecular states of ions in protein cavities can be a mechanism of non-specific magnetobiological effects. Recent works by the same author describe the level mixing mechanism (LMM) [76,307]. The mechanism is based on the inhomogeneous precession and thermal relaxation of the magnetic moment in the MF. The primary sensors of weak magnetic fields in the LMM model can be molecules or molecular groups in nucleic acids and proteins that possess magnetic spin and make rotational motions. Such targets in hypomagnetic conditions will stop their rotation, while in TVMFs they will precess.

The possible explanations of interactions of MFs with rotating molecules or individual molecular groups are described in detail in works [305,316,317]. Potential targets in this case are non-thermal rotations of RNA, DNA, enzymes, and synthesized proteins [305,318]. A mathematical modeling method shows the basic possibility of inducing rotational vibrations in the DNA molecule under the action of an external force of an electromagnetic nature. It has been found that the frequency of such specific oscillations of a DNA molecule depends on the sequence of nucleotides [319]. The precession of a molecule becomes uneven in an AMF or slows down in a weakened TVMF or SMF [241,305]. A change in the rate of rotation of a molecule and its magnetic moment finally leads to its conformational changes and signal transduction to the level of biochemical reactions [320]. According to calculations, in the case of molecular rotations, the *kT* problem is solved [305].

Recently, oscillatory biochemical processes have been proposed as a target for MFs to achieve resonance-like responses of biological systems to ELF-MFs [48]. For example, the cycles of ROS generation/removal in mitochondria and due to changes in superoxide dismutase activity may be a potential target [247,248]. The concept of oscillating biochemical processes combines several described mechanisms of the magnetobiological action of ELF-MFs. In particular, the targets of the MFs are radical pairs, and the main mechanism is resonant-like phenomena. Radical targets must be generated and used in cells. If the frequency of the oscillation of the target concentrations coincides with that of an ELF-MF, a biological effect is realized. Only a fraction of radical pairs can do this. The coincidence between the oscillations of the radical pair generation rate and the oscillations of the ELF-MF needs to induce biological effects. Synchronization of ELF-MF frequency with the frequency of chemical oscillations provides an “effective” MF for radical pairs in a portion of chemical oscillators. The ratio of triplet and singlet yields for this portion of oscillators will differ from the state for the rest of the oscillators throughout the whole ELF-MF exposure due to the non-linear dependence between the triplet and singlet yields and MF intensity [321]. The disappearance of the biological effect at a changed non-resonant LFMF frequency can be a consequence of the inability to maintain an “effective” state of the portion of the biochemical oscillators throughout the ELF-MF exposure. It ensures the appearance of frequency windows of magnetobiological effects [48]. The biologically effective amplitude of the ELF-MF exists for a specific radical-pair reaction. A change in ELF-MF amplitude can shift the MF intensity values to the area of linear dependence, which leads to the absence of a biological effect. It explains the amplitude windows of the LFMF efficiency [322].

## 5. Dependence of Quantitative Characteristics of Biological Effects of ELF-MFs on Their Frequency, Induction, and Duration

**Figure 6:**
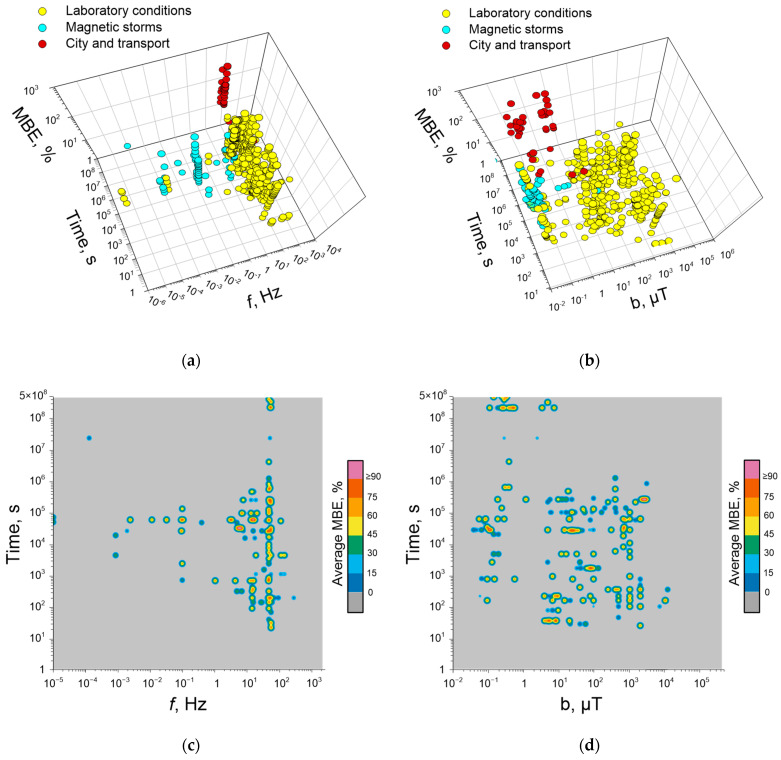
Estimation of the magnitude of magnetobiological effects (MBEs) from the amplitude–frequency characteristics and exposure time of an ELF-MF. Three-dimensional dot-plots (**a**,**b**) and 3D contour plot (**c**,**d**) distributions of the magnitudes of biological effects from the frequency values f (**a**,**c**) of induction b of the variable component (**b**,**d**) over time. Each point is a separate frequency/time/effect or induction/time/effect value reported in the literature. The MBE was calculated as the ratio of each parameter after magnetic exposure to the initial value of this parameter (taken modulo), expressed as a percentage. (**a**,**b**) The color of the dots shows the ELF-MF source: yellow—laboratory conditions, blue—magnetic storms, red—background fields of cities and transport. (**c**,**d**) The color indicates MBE values: red—high values, blue—low (References in Table 1 and Table 2). These images were created using the color lookup of the table panel plugin developed by Patrick Pirrotte and Jerome Mutterer (https://imagej.net/ij/ij/plugins/lut-panel.html, accessed on 15 October 2023) based on a color-blind friendly set proposed by Masataka Okabe and Kei [326]. The literature data used in the calculations and figures can be found in the Supplementary Materials.

**Figure 7:**
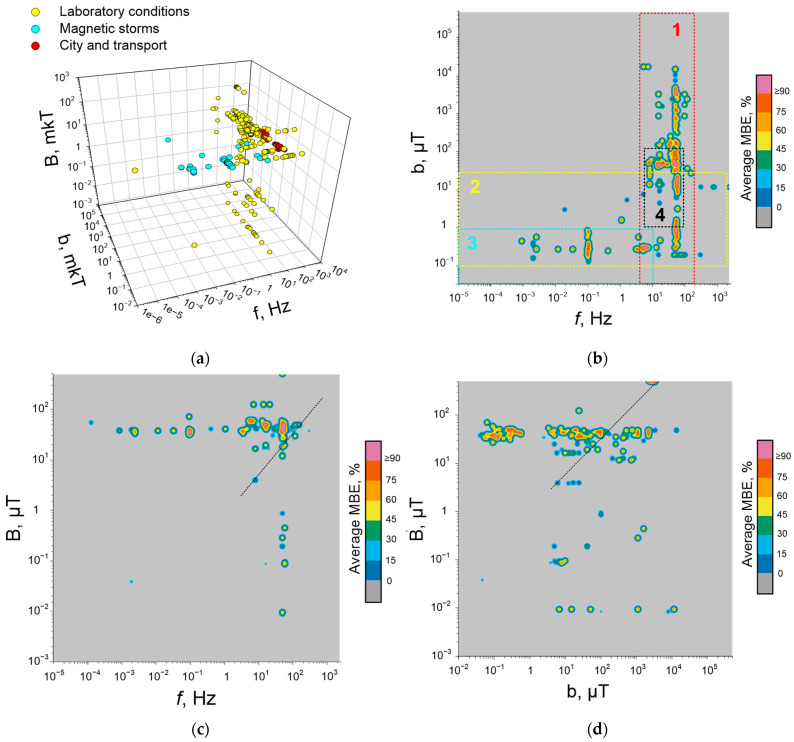
Estimating the magnitude of biological effects of ELF-MFs on frequency, AMF induction, and SMF. Dot-plots of the distribution of work according to the applied values of frequency (*f)* and inductions of AMF (b) and SMF (B) (**a**). Each point is a separate *f*/b/B value reported in the literature. Three-dimensional contour plots of the distribution of the magnitude of magnetobiological effects (MBE, %) by *f*/b (**b**), *f*/B (**c**), or b/B (**d**) values. The biological effect was calculated as the ratio of each parameter after magnetic exposure to the initial value of this parameter and expressed as a percentage. (**a**,**b**) The color of the dots shows the ELF-MF source: yellow—laboratory conditions, blue—magnetic storms, red—background fields of cities and transport. (**b**). The areas highlighted by rectangles show amplitude–frequency “windows”: 1—industrial frequencies and their harmonics and subharmonics, 2—background ELF-MFs of cities and transport, 3—geomagnetic storms, and 4—area of cyclotron resonances. (**c**,**d**) The dashed lines show examples of *f*/B and b/B ratios consistent with the Blanchard and Blackman model for describing cyclotron resonances for Li cyclotron frequencies (References in Table 1 and Table 2). These images were created using the color lookup of the table panel plugin developed by Patrick Pirrotte and Jerome Mutterer (https://imagej.net/ij/ij/plugins/lut-panel.html, accessed on 15 October 2023) based on a color-blind-friendly set proposed by Masataka Okabe and Kei [326]. The literature data used in the calculations and figures can be found in the Supplementary Materials.

The magnitude of the change in biological parameters depends on the physical characteristics of the applied ELF-MF in a complex manner (Figure 6 and Figure 7, Tables S1 and S2). Often, biological effects appear only in “windows” of frequency and induction values [323,324,325].

This is especially clearly seen in works where an increase in MF induction leads to the alternate disappearance and restoration of the effect [327,328].

We attempted to assess the diversity of “windows” by constructing 3D maps of the distribution of magnetobiological effect (MBE) values depending on the frequency of the ELF-MF and the duration of magnetic exposure (Figure 6a), AMF induction of ELF-MF and duration (Figure 6b).

We discovered the following patterns for biologically active ELF-MFs of different natures. Most ELF-MFs generated in laboratory conditions have a relatively narrow frequency range (9–60 Hz) and a wide AMF induction range (10^−2^–10^5^ μT).

For ELF-MFs during magnetic storms, the situation is the opposite. The frequency range is wide (10^−4^–10^1^ Hz) and the induction range is narrow (~1 × 10^2^–5 × 10^2^ nT). Background ELF-MFs of cities and transport are usually limited in amplitude from 30 to 100 μT and are realized in a wide frequency range from 10^−4^ to 10^3^ Hz and higher. It is noteworthy that the ELF-MF generated in the laboratory only partially “overlaps” the spectral content of ELF-MF magnetic storms and the background MF of cities and transport. In the case of magnetic storms, there is “no overlap” in frequencies; in the case of background anthropogenic fields, there is “no overlap” in time. Epidemiological effects are detected over several years [69,70]. Laboratory studies rarely exceed the time threshold of 1–2 days of exposure (~10^5^ s, Figure 6). Studies of several weeks or months are very rare [222]. This limits studies to the frequency range (10^−4^–10^−3^ Hz) characteristic of magnetic storms. On the other hand, long-term experiments are conducted on animals, and this limits the transfer of the obtained MBEs to humans.

Apart from epidemiological studies, the effects of ELF-MFs are weakly time-dependent and highly dependent on frequency (*f*) and inductions of AMFs (b) and SMFs (B) (Figure 6c,d). Given this, we estimated the distribution of MBE values from the combination of *f*/b, *f*/B, and b/B (Figure 7). The areas of manifestation of biological effects in this case turned out to be quite narrowly localized.

The first and most “obvious” range lies in the frequency and induction limits *f*(50–60 Hz) and corresponds to industrial MFs. Formally, this range can be divided into two parts: strong ELF-MF effects *f*(10–300)B(>10 µT) industrial frequencies and their harmonics and subharmonics (fields with such induction are rarely encountered in everyday life and are used in laboratory experiments, for example, to test theories about cyclotron resonances (Figure 7b (1)) [27,28,261]; weak MFs (<10 µT) of the same frequency range are often encountered in everyday life. In addition, in everyday life, we are surrounded by urban background MFs mainly consisting of noise from electrical equipment, transportation, etc. [19,26,27,28,56].

The third range is the amplitude–frequency characteristics of magnetic storms *f*(0.001–30 Hz)B(80–900 nT) [26,27,28,29,30].

The fourth range corresponds to cyclotron resonances of atoms of biogenic elements, in particular for B = 43 μT, ^6.9^Li = 94.8 Hz, ^23^Na~28.6 Hz, ^24.3^Mg~54.1 Hz, ~54 Hz, ^41^K~16.9 Hz, ^42^Ca~31.3 Hz, and ^64^Zn~17.0 Hz [187]. In some studies, authors were able to estimate the b/B ratio of biologically active ELF-MFs [149,165,187,278]. We found an example of *f*/B and b/B ratios of biologically active ELF-MFs, which seem to agree with the theoretical model (see above) based on cyclotron resonances (Figure 7d).

The Schumann resonance phenomenon is described at frequencies 7.8, 14.1, and 20.3 Hz. It is the phenomenon of the formation of standing electromagnetic waves of ultra-low frequencies between the Earth’s surface and the ionosphere [283,284]. As stated above, the Schumann resonance EMFs have an extremely small ~1 pT induction [117]. However, biological effects are found at Schumann resonance frequencies 7.8, 14.1, and 20.3 Hz [117,226,292]. Examples of biological effects of GMF fluctuations on Schumann resonance frequencies were described in Section 4.

The sub-range *f*(10^−3^–10^−2^) may be interesting. These frequencies correspond to the frequencies of slow biorhythms, in particular, oscillations of brain potentials recorded by EEG [329,330].

We assume that the study of the biological effects of ultra-low-frequency MFs with frequencies of 10^−4^–10^1^ Hz is promising. Therefore, this range includes the effects of magnetic storms, anthropogenic MFs, and areas of cyclotron resonances, as well as several low-frequency biorhythms.

## 6. Influence of Environmental Factors

Magnetobiological effects depend on many factors. They can be conditionally divided into two large groups: physical and biological. Among physical factors, it is possible to note the dependence of effects on the amplitude and frequency of TVMFs, the dependence on the induction and directivity of direct MFs, the dependence on the polarization of the electric and MF intensity vector, and the dependence on amplitude modulation. The influence of the concentration of Ca^2+^ ions in the surrounding solution on the expression of the biological effect of the *f*(16)b(20u9)B(52)t(48 h) field on the mobility of diatom algae is described. The dependence had a dome-shaped form with a maximum concentration of 0.25 mM [149]. Dependence on the time of exposure and the ambient temperature can be separately noted [221]. Differences in cell type, genetic and epigenetic, initial state of cells, and cell cycle phase may be attributed to biological factors.

The influence of some physical factors can be traced back to microwave radiation. Although this review concentrates on low-frequency MFs, in this section we will allow ourselves to cite the bioeffects of microwaves since the influence of some physical parameters of radiation is difficult to visualize for low-frequency MFs. For example, the dependence of resonance-like effects on the type of polarization (circular or linear) or even the polarization direction (left or right) was found for different cell cultures and different *E.coli* strains [331]. The interesting phenomenon of resonance-like peak broadening of the magnetobiological effect with increasing radiation power was found in another study, and for millimeter fields [332]. For high-frequency MFs, magnetobiological effects are often reported when the signal is modulated by amplitude or frequency. Many examples of modulation, mainly by amplitude modulation, are given in the review [333]. Exposure to low-frequency microwave modulation resulted in changes in the EEG of human subjects [334].

Interesting work was carried out on the effect of microwaves on *E. coli* strain K12 N99 and two lysogenic strains with added bacteriophages [335]. The addition of bacteriophages shifted the resonance-like peak in the microwave regions of 41 and 51 GHz. The degree of the shift depended on the length of the DNA. This phenomenon raises the question of DNA as the primary target of MFs of this range. For low-frequency MFs, DNA can also serve as a target [335]. The DNA double-strand breaks were demonstrated under the influence of sufficiently strong (7 mT) 60 H MFs *f*(60)b(7 mT) [336]. In addition, the authors did not observe the formation of reactive oxygen species. The magnitude of the magnetobiological effect depends on the mode of field generation—continuous or pulsed. Pulsed mode ELF-MF *f*(50)b(1 mT) on/off 5 min/10 min during 24 h induced single- and double-stranded DNA breaks in human diploid fibroblast cells [337]. At the same time, the genotoxic effects of MF are not observed in other studies with a continuous mode of MF generation [173,338].

The influence of the initial state of the biological object can also be noted among the biological factors. For example, the magnitude and direction of the biological effects of a 50 Hz field of different amplitude depended on the initial state of lymphocyte chromatin, which in turn depended on the donor and the temperature before and during MF exposure [42]. The possible influence of epigenetic profile on the magnetobiological effect was described. An epigenetic profile is known to be sensitive to environmental conditions [125]. Exposure to low-frequency MFs affected the profile of histone and DNA modifications, which were stochastic and appeared to be manifested in a genomic context-dependent manner. Another example of the dependence of bioeffects on the initial state of a biological object is the logarithmic or stationary phase of cell growth in *E. coli* cultures [228]. As mentioned above for microwaves, the effects for low-frequency fields were also dependent on bacterial strain [30,187]. Differences in effects were also found as a function of exposure duration, cell density, and post-exposition time [339].

For eukaryotic cell lines, the effects of ELF-MFs with close amplitude–frequency characteristics and durations can depend significantly on the specific cell line. For example, ELF-MF *f*(50)b(1000–2000)B(40–50)t(24 h) accelerated the differentiation of neural stem/progenitor cells of newborn mice but did not affect the differentiation of cell lines U251, A172, SH-SY5Y, and primary cultured neurogenic cells from rat embryo astrocytes and microglia [175,181,182,229]. The dependence of ELF-MF effects on time after exposure can have different characteristics. The degree of manifestation of biological effects can be either monotonically increasing/decreasing with time or have a complex form of increasing and decreasing [142,147,160,221]. In particular, it has been shown in fruit flies that the effects of magnetic exposure can be manifested in subsequent generations, F1, F2, etc. [158]. At the same time, during the transition from F1 to F2, a change in the direction of the biological effect and the degree of its manifestation is possible [158].

Different tissues have different ”sensitivities” to ELF-MFs, even within the same organ. For example, hippocampal neurons respond to *f*(50)b(1000)B(0.001)t(10 h) with 20–35 times greater Ca^2+^ release than cortical and cerebellar neurons [136].

## 7. Biological Effects of Extremely-Low-Frequency Electrical Fields

The electric and magnetic components of high-frequency (≫100 Hz) EMFs are connected by the Poynting vector in the zone far from the emitter (*r* ≫ *λ*, where *r* is the distance to EMF source and *λ* is a wavelength) [340,341]. In this case, the electromagnetic wave is formed, and measurements of one component automatically give the value of the second component. The magnetobiological effects of high-frequency EMFs should be considered through the prism of the simultaneous action of both magnetic and electric components. For low-frequency EMFs (<100 Hz), we are always in the near zone (*r* ≥ λ or *r* < *λ*) where the electromagnetic wave is just forming. In the low frequencies (ULF and ELF) the connection/dependence between the E and B components, depending on their time derivatives, is weak, and for this reason, they are measured separately.

Historically and recently, biological effects of EFs and MFs were often considered together [118,342,343]. We suppose that the effects of electric (EFs) and magnetic fields (MFs) in this frequency range should be considered separately. EF- and MF-dependent effects were described in some works. For example, the MF had a greater effect on protecting chicken embryos from lethal hypoxia than the electric component of EMFs [155]. There are also studies on the biological effects of low-frequency and constant EFs. They are briefly described in this section.

First, we should imagine what natural conditions surround us in terms of electrostatic or low-frequency electric fields. The GMF has very conservative values for ~30–60 µT constant component and 500 nT low-frequency (<1 Hz) variations. The range of natural geo-electric field (GEF) variations is quite large. The GEF strength varies from ~100 V/m near the Earth’s surface on a calm day to >10 kV/m before a thunderstorm [5,344,345]. Anthropogenic sources of EFs can be much more intense than natural ones. For example, clothing worn on the human body can generate electrostatic fields of >100 kV/m [346], and 600 kV power lines can generate fields of >15 kV/m at distances of up to 30 m [347].

Based on mathematical modeling, EFs do not penetrate deep into biological tissues, unlike constant and ELF-MFs [348]. However, living tissue is too complicated to be simulated by inanimate materials. ELF E-fields not only penetrate enough, but in addition, they can act on skin cells and have profound biological effects on the whole organism. Moreover, they can enter the living tissue through nerve terminals on the skin. Effects of very weak ULF/ELF EFs on living tissue have been recorded [349]. Several studies have found more correlation with the electric than with the magnetic component of power frequency EMFs [234,350].

This specificity of EF-induced effects is reflected in biological effects. Effects of EFs appear to be maximal in animals with sensitive surface-sensing organs. For example, even small, extremely low-frequency (0.1–50 Hz) and low-voltage (0.024–0.3 V) EFs induced an avoidance response in lake sturgeon [351]. Rodents can also be included in this range. For example, exposure to 50 Hz 10 kV/m for 60 min suppressed the stress response, causing an increase in glucocorticoid levels in immobilized mice, and slightly increased glucocorticoid production in the absence of stress [352]. Increases in the stress response (in adrenocorticotropic hormone, glucose, lactate, and pyruvate) with hourly exposure to 50 Hz 17.5 kV/m EF for 60 min were also observed in stressed rats [353]. In chronic exposure of six generations of mice, corticosterone levels were significantly higher in exposed individuals at 10 kV/m EF. A low-frequency electric field up to 100 V/m does not appear to cause any observed effects in rats [354].

Strong 50 Hz EFs with an intensity between 500 and 5000 kV/m can have a significant impact on small insects. This effect can be fatal, either directly, as observed in fruit flies [355], or indirectly through aggressive behavior, as seen in bees [356].

The impact of constant and low-frequency electric fields on humans is also described in the literature. Exposure to 30 kV/m and 50 Hz EF increased alpha and theta EEG rhythms and a low-frequency HRV component [357,358]. However, some studies have not demonstrated any effects of such fields [359,360].

It is worth noting that the observed effects in this area are extremely controversial. This is especially evident in the example of EF effects on microorganisms. On the one hand, there are studies in which EFs of 4–6 V/m intensity with a frequency of 50 Hz led to an increase in metabolism and division of microorganisms [361]. On the other hand, there are attempts to use ELF-EFs for low-temperature preservation of products, i.e., protection against microorganisms [362].

## 8. Conclusions and Prospects

ELF-MFs with a frequency of <1 kHz have a wide range of biological effects on living systems. These fields include fluctuations of the GMF during a magnetic storm and background TVMF generated by electrical equipment, transport, etc. Among the main effects of magnetic storms on humans, changes in the cardiovascular system are primarily noted. Anthropogenic ELF-MFs affect the functioning of the cardiovascular system and may also be associated with the risk of developing some kinds of cancer. ELF-MFs studied in the laboratory had the most diverse effects on the circulatory, nervous, immune, endocrine, and musculoskeletal systems of humans and animals, as well as on plants and insects. We attempted to search for patterns connecting the MF spectral content and the level of the biological effect. It was found that most of the effects are localized in amplitude–frequency “windows”; maxima are observed in the areas of cyclotron resonances of mono- and divalent ions, industrial MFs, and magnetic storms. The analysis approach we used can be expanded by introducing additional parameters (field direction, presence of rotation, the shape of a single signal, etc.). The results obtained may be of fundamental value and find practical application in biology, medicine, and agriculture.

## Data Availability

The data presented in this study are publicly available and contained in Supplementary Materials.

## Supplementary Materials

The following supporting information can be downloaded at: https://www.mdpi.com/article/10.3390/biology12121506/s1, Table S1: The relationship between magnetic storms characteristics and magnetobiological effects; Table S2: The relationship between characteristics antropogenic ELF-MF and its magnetobiological effects. b and B are the amplitudes of TLVF oscillations and SMF trspectively in μT, *f* is the frequency in Hz, t—total exposure duration in seconds. “Effect % modulus” is value of magnetobiological effect taken modulo.

## Abbreviations

ELF-MF	extremely-low-frequency magnetic fields
EMF	electromagnetic fields
EMF	electromagnetic fields
*f*	frequency
GMF	geomagnetic field
MF	magnetic fields
SJR	scientific journal ranking
SMF	static magnetic fields
TVMF	time-varying magnetic fields
